# A marmoset brain cell census reveals regional specialization of cellular identities

**DOI:** 10.1126/sciadv.adk3986

**Published:** 2023-10-12

**Authors:** Fenna M. Krienen, Kirsten M. Levandowski, Heather Zaniewski, Ricardo C.H. del Rosario, Margaret E. Schroeder, Melissa Goldman, Martin Wienisch, Alyssa Lutservitz, Victoria F. Beja-Glasser, Cindy Chen, Qiangge Zhang, Ken Y. Chan, Katelyn X. Li, Jitendra Sharma, Dana McCormack, Tay Won Shin, Andrew Harrahill, Eric Nyase, Gagandeep Mudhar, Abigail Mauermann, Alec Wysoker, James Nemesh, Seva Kashin, Josselyn Vergara, Gabriele Chelini, Jordane Dimidschstein, Sabina Berretta, Benjamin E. Deverman, Ed Boyden, Steven A. McCarroll, Guoping Feng

**Affiliations:** ^1^ Department of Genetics, Harvard Medical School, Boston, MA 02115, USA.; ^2^ Stanley Center for Psychiatric Research, Broad Institute of MIT and Harvard, Cambridge, MA 02142, USA.; ^3^ McGovern Institute for Brain Research, Department of Brain and Cognitive Sciences, Massachusetts Institute of Technology, Cambridge, MA 02142, USA.; ^4^ Department of Brain and Cognitive Sciences, Massachusetts Institute of Technology, Cambridge, MA 02139, USA.; ^5^ Howard Hughes Medical Institute, Cambridge, MA 02139, USA.; ^6^ Princeton Neuroscience Institute, Princeton University, Princeton, NJ 08544, USA.; ^7^ Center for Mind/Brain Sciences, University of Trento, Piazza della Manifattura n.1, Rovereto (TN) 38068, Italy.; ^8^ Basic Neuroscience Division, McLean Hospital, Belmont, MA 02478, USA.; ^9^ Department of Psychiatry, Harvard Medical School, Boston, MA 02115, USA.

## Abstract

The mammalian brain is composed of many brain structures, each with its own ontogenetic and developmental history. We used single-nucleus RNA sequencing to sample over 2.4 million brain cells across 18 locations in the common marmoset, a New World monkey primed for genetic engineering, and examined gene expression patterns of cell types within and across brain structures. The adult transcriptomic identity of most neuronal types is shaped more by developmental origin than by neurotransmitter signaling repertoire. Quantitative mapping of GABAergic types with single-molecule FISH (smFISH) reveals that interneurons in the striatum and neocortex follow distinct spatial principles, and that lateral prefrontal and other higher-order cortical association areas are distinguished by high proportions of *VIP*^+^ neurons. We use cell type–specific enhancers to drive AAV-GFP and reconstruct the morphologies of molecularly resolved interneuron types in neocortex and striatum. Our analyses highlight how lineage, local context, and functional class contribute to the transcriptional identity and biodistribution of primate brain cell types.

## Teaser

Adult primate neurons are imprinted by their region of origin, more so than by their functional identity.

## INTRODUCTION

The vertebrate brain is a complex tissue composed of highly heterogeneous and spatially regionalized cell types (*1*–*8*). The molecular composition of brain cells is influenced by many factors, including the cells’ function, developmental and phylogenetic history, and regional context; each factor contributes in some part to overall cellular identity. The marmoset, a small, New World monkey, diverged from humans ~45 million years ago and has a near-lissencephalic brain that is the size of a squirrel. Despite its small size, the marmoset brain retains many of the structural and functional innovations that have accumulated along the primate lineage (*9*–*14*). Because of its small size, the marmoset brain also offers practical advantages for studying primate cell type diversity: Sequencing 1 million neurons would achieve coverage of 0.001% of the total neurons in the marmoset brain, yet would represent far lower coverage (0.00001%) of the neuronal composition of the human brain (*15*). Previous single-cell sequencing studies of the marmoset brain focused on single brain regions (*16*, *17*) or on specific cell classes across regions (*10*, *18*). Inclusion of both closely and distantly related brain structures and cell types can yield insights into the sometimes surprising developmental and ontological relationships between them (*19*).

To better appreciate what shapes a primate brain cell’s identity, we conducted a census of brain cell types of the adult marmoset using single-nucleus RNA sequencing (snRNA-seq). We generated snRNA-seq (10x Genomics 3′ v3.1) data from 2.4 million unsorted brain cell nuclei across 8 neocortical and 10 subcortical locations from 10 young adult marmosets (4 male, 6 female) and resolved clusters from all major neuronal and nonneuronal cell classes. snRNA-seq data were generated as part of the Brain Initiative Cell Census Network (BICCN; RRID:SCR_015820) and are available on the BICCN Data Center (RRID:SCR_022815; https://biccn.org/data) as well as the NeMO archive (RRID:SCR_016152, https://scicrunch.org/resolver/RRID:SCR_016152; https://doi.org/10.1101/2022.10.18.512442). Processed data can be accessed and explored through the cellxgene data repository (RRID:SCR_021059).

All neuron-containing brain structures in the central nervous system (CNS) have both excitatory and inhibitory neuronal populations, although the proportions and degree of developmental relatedness between these two populations vary by structure. In the neocortex and other telencephalic structures of vertebrates, distinct populations of adult neurons are often categorized by their primary neurotransmitter status, typically either inhibitory [γ-Aminobutyric acid (GABA)-ergic] or excitatory (glutamatergic). In other brain structures, primary neurotransmitter usage appears less essential to a cell type’s identity. We found that the transcriptional identities of excitatory and inhibitory neurons within telencephalic brain structures segregate strongly, consistent with previous studies in other mammalian species (*4*, *5*, *17*, *20*). In contrast, there is much greater transcriptional similarity between GABAergic and glutamatergic neuronal types in nontelencephalic compartments. Moreover, few gene expression distinctions present in telencephalic glutamatergic neurons are shared with glutamatergic neurons in nontelencephalic brain regions.

While primary neurotransmitter status did not drive transcriptional similarity between neurons, their brain structure of origin did: The adult transcriptomic identity of most neuronal types appears to be shaped more by their developmental or regional identity than by their neurotransmitter signaling repertoire. In line with previous findings in diverse mammalian species (*2*, *5*, *18*), we also found regional heterogeneity in nonneuronal cells, particularly astrocytes.

The neocortex is parcellated into a mosaic of functional areas. While our snRNA-seq results and others (*2*, *10*, *21*) indicate that cell type proportions vary considerably between discrete cortical areas, spatially resolved approaches are better suited for appreciating large-scale trends and finer discontinuities in the biodistribution of cell types (*22*). Although the marmoset brain is small, potentially owing to secondary dwarfism of Callitrichids (*23*), its neocortex is still highly functionally parcellated and retains primate-specific specializations (*9*–*14*). Moreover, the small size of the marmoset neocortex enables quantitative, spatial mapping of cell types across the entire anterior-posterior extent within single sections. We therefore used single-molecule fluorescence in situ hybridization (FISH) (smFISH) to spatially profile major interneuron types across marmoset striatum and neocortex. We found that in the striatum, major interneuron types are distributed as medial-lateral gradients. In contrast, the marmoset neocortex has a complex topography of interneuron concentrations that do not follow a single spatial axis. Lateral prefrontal areas, in particular, which have undergone major expansion in primate evolution (*24*), are typified by higher proportions of *VIP*^+^ and *LAMP5/ID2*^+^ interneurons and lower proportions of *SST*^+^ interneurons relative to all other neocortical areas.

As cellular morphology is critical to neuron function and type definition (*25*–*28*), we sought to provide a compendium of morphologically reconstructed interneurons in striatum and neocortex. We reconstructed a set of molecularly characterized interneurons in marmosets that had received systemic delivery of an adeno-associated virus (AAV) carrying a reporter (AAV9-hDlx5/6-GFP-fGFP) under an interneuron-selective regulatory element (*29*). Reconstructions are available for download from the Brain Image Library (BIL; https://doi.org/10.35077/g.609). As AAV9-hDlx5/6-GFP-fGFP did not label a primate-specific, *TAC3*+ striatal interneuron type that we previously discovered using snRNA-seq (*10*), we acquired single-nucleus ATAC-seq (snATAC-seq) data from marmoset striatum and used *TAC3* interneuron–specific enhancers to successfully express green fluorescent protein (GFP) in these cells, enabling their morphological reconstruction.

Comprehensive cell type atlases of mouse (*1*, *4*, *5*, *30*) and regional sampling across humans and nonhuman primates brains (*2*, *18*, *21*, *31*) have begun to facilitate comparative analysis of brain cell type features (*10*, *17*, *32*, *33*). Together, our census of major transcriptomically defined brain cell types and quantitative mapping of interneuron biodistribution and morphology in the marmoset serve as a resource for the primate neuroscience community and for studies of cell type development and evolution.

## RESULTS

### A transcriptomic census identifies major marmoset brain cell types

**Fig. 1.**
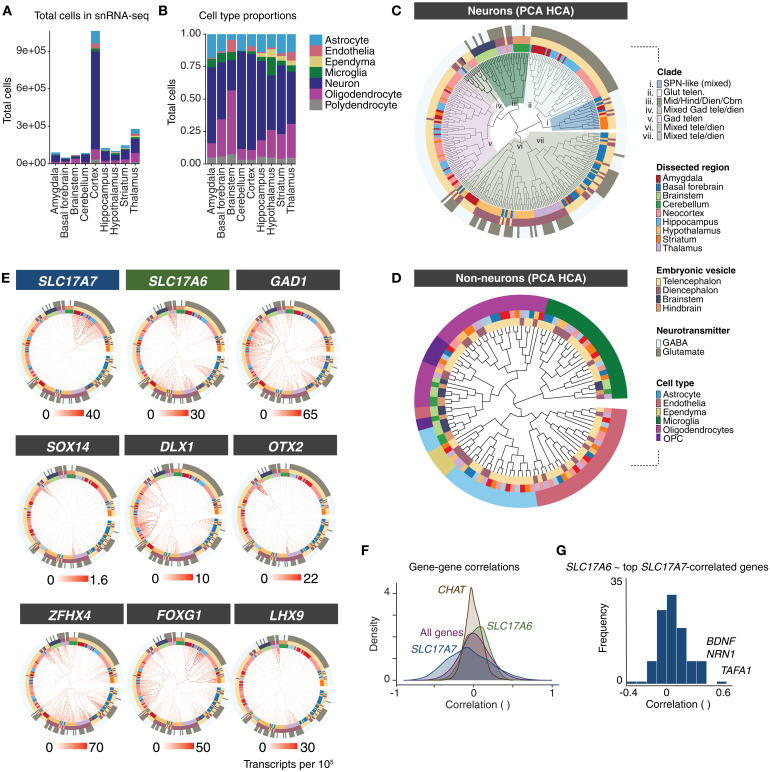
Single-nucleus RNA sequencing of marmoset brain. (**A**) Number of nuclei per brain structure. Colors indicate cell classes. (**B**) Proportions of cell classes across brain structures. (**C**) Neurons in each dissected region are clustered separately, and then pseudo-bulked “metacells” of all 288 clusters are arranged in the dendrogram using hierarchical clustering of top 100 PCA scores of expressed genes. Outer rings colored by: dissected subregion (inner ring), cephalic vesicle (second ring), and major neurotransmitter usage (outer ring). Seven major “clades” are colored and labeled according to cell type and regional composition of clade. (**D**) Same hierarchical clustering procedure as (C) but for nonneuronal cell types. Outer ring colors indicate major nonneuronal cell class. Inner ring colors indicate region dissection and vesicle; colors as in (C). (**E**) Expression of markers for glutamatergic neurons (*SLC17A7*, *SLC17A6*), GABAergic neurons (*GAD1*), and TFs (*FOXG1*, *ZFHX4*, *SOX14, DLX1, OTX2, LHX9*) are plotted as heatmaps on dendrogram shown in (C). These TFs are largely restricted to specific clades or are associated with particular cephalic vesicles. (**F**) Gene-gene correlation (Spearman tau) distributions across all neuron clusters in (C) for *SLC17A7*, *SLC17A6*, and *CHAT* (marker for cholinergic neurons) as well as background (all genes). The distribution of pairwise correlations to *CHAT* had a lower SD (mean *r* = 0.002, SD = 0.116) relative to baseline gene-gene correlations (mean *r* = 0.02, SD = 0.199; *F*_5078,17315809_ = 0.33, *P*_adj_ < 1 × 10^−15^). In contrast, pairwise correlations to *SLC17A7* were much broader relative to the background distribution of all gene-gene correlations. (**G**) Distribution of cross–cell type correlation to *SLC17A6* of genes most correlated with *SLC17A7* (top 116 genes with Spearman tau > 0.5 to *SLC17A7*).

We acquired snRNA-seq data from 18 brain regions collected using 10x Genomics 3′ 3.1 chemistry across 10 young adult marmosets (ages 2 to 5 years) as well as a small dataset from the lateral prefrontal cortex (PFC) of two aged animals (Fig. 1, fig. S1, and table S1). The number of donors per brain structure varied (min = 2, max = 10; fig. S1), as did the cell sampling rate per brain structure (Fig. 1A, fig. S1A, and table S2). Neocortex was the most comprehensively sampled in terms of total numbers, donors, and regional dissections (Fig. 1A, fig. S1A, and table S2). We acquired data from cerebellum, brainstem, hypothalamus, thalamus, amygdala, striatum (separate dissections for caudate, putamen, and nucleus accumbens), hippocampus, basal forebrain, and neocortex. We did not attempt to dissect each brain structure in its entirety; this census should be viewed as containing representation from each of these structures, but not exhaustive profiling thereof. For example, the hypothalamus and brainstem contain tremendous cellular heterogeneity that varies across relatively small spatial scales due the presence of specialized brain nuclei and rare cell types (*1*, *3*, *34*); our census only sampled small portions of these structures and is not comprehensive. Within the neocortex, we separately sampled eight neocortical locations (prefrontal, temporal pole, S1, M1, A1, V2, V1, and lateral parietal association), and within PFC, from four distinct subdivisions (dorsolateral, medial, orbital, and ventral) (fig. S1, B and C).

Nuclei from each brain structure were pooled across donors and analyzed to identify major cell types and their proportions by brain structure (Fig. 1, B to D). Using linear discriminant analysis [scPred; (*35*)] trained on a supervised set of cell class labels, we identified and discarded low-quality cells and doublets, and assigned each nucleus to its probable major type—astrocyte, endothelia, ependyma, microglia/macrophage, neuron, oligodendrocyte, or oligodendrocyte precursor cell (OPC). After major cell type assignment, nuclei were clustered by brain structure to reveal subtype diversity within each major class. We used a previously described clustering pipeline (*4*) based on independent components analysis (fastICA); each clustering analysis involved additional curation of doublets and outlier cells, followed by a second round of subclustering of major clusters. At each of these curation stages, independent components (ICs) that loaded on individual donors or batches were excluded and reclustering was performed to attenuate donor and batch effects on clustering results. For each cluster, a “metacell” (conceptually similar to “pseudo-bulking”) was generated by summing transcript counts of individual cells of each cluster together, followed by scaling and normalizing to 100,000 transcripts per nucleus. These regional and cell type–resolved metacells were the starting point for most cross-region analyses. The final snRNA-seq dataset size after curation contained 2.01 million cells (table S2).

### Hierarchical clustering reveals regional specialization of marmoset brain cells

Cell types are identified based on many factors including their function, morphology, developmental origin, and regional context (*36*–*38*). We studied how transcriptional profiles related to each other across 288 neuronal clusters (metacells) that were sampled across all brain structures. Individual replicates and other variables (age, sex) were generally proportionately represented across resolved clusters, with several exceptions that were likely mainly to differences in dissection across donors (figs. S1 and S2). We used hierarchical clustering to situate the neuronal types on a dendrogram computed using distances calculated from the top 100 principal component (PC) scores across neuron types (HCA-PCA; Fig. 1C and table S2). Most telencephalic types (neocortex, amygdala, hippocampus, striatum) clustered distinctly from diencephalic and hindbrain types, indicating that developmental lineage continues to shape the transcriptional identity of adult primate brain cell types. However, of the seven major clades, four contained mixtures of cell types at the terminal (leaf level) originating from distinct cephalic compartments. For example, basal forebrain neuron types intermingled with hypothalamic types, suggesting closer transcriptional similarity of two structures that are typically considered to occupy distinct cephalic compartments.

We next conducted HCA-PCA clustering on 111 nonneuronal metacells curated from all sampled brain structures (Fig. 1D). Major clades of this dendrogram reflected nonneuronal class; hematopoietic lineages (microglia and brain-resident macrophages) were the most transcriptionally divergent from the other nonneuronal types. Ependymal cells, detected only in structures that border the ventricles (particularly numerous in thalamus, striatum, and hypothalamus dissections), were transcriptionally most similar to astrocytes (Fig. 1D). As prior transcriptomic studies in mouse (*1*, *5*), marmoset (*18*), and human brains (*2*) have also shown, astrocytes were particularly heterogeneous across major subdivisions (fig. S2, A to H). We validated the differential expression of two genes that distinguished astrocytes in the cortex (telencephalic origin, *FOXG1*) and thalamus (diencephalic origin, *SPARC*) in one marmoset using smFISH (fig. S2, F to H).

### Neurotransmitter usage is not strongly associated with transcriptomic identity

The neurotransmitters used by neurons are central to their function, and neurons are often classified by their primary neurotransmitter. However, synthesis of the major neurotransmitters—glutamate (in mammals, excitatory) and GABA or glycine (inhibitory)—requires only a modest number of genes, and GABA itself is synthesized from glutamate (*39*). As neuronal transcriptomes tend to show strong influences of their regional or developmental origin (Fig. 1C), the extent to which neurotransmitter utilization is strongly associated to the transcriptomic identity of neuron types may differ by brain structure. Transcriptomically defined neuron types in the neocortex and other telencephalic structures hierarchically partition into GABAergic and glutamatergic types (*5*, *8*, *17*), which reflects both their distinct developmental origins and their distinct neurotransmitter repertoire. In other brain structures such as the hypothalamus, the distinction between neurons using GABA or glutamate is much less clear at the transcriptional level (*1*, *3*), likely as a consequence of shared developmental lineage.

To determine how neurotransmitter identity was associated with a general transcriptional identity across all neurons expressing the same neurotransmitter, we examined expression of genes encoding the most prevalent vesicular glutamate transporters (*SLC17A6, SLC17A7*) and GABA synthesis enzymes (*GAD1, GAD2*) (Fig. 1, C to F). If primary neurotransmitter usage was strongly associated with the expression of many other genes, we would expect that neurons expressing the same neurotransmitter-associated genes (GABAergic or glutamatergic) would preferentially group together regardless of other factors (such as developmental origin). However, we did not find evidence for strong global transcriptomic identities of GABAergic and glutamatergic neurons. Neuronal types from each set were distributed across the tree, suggesting widely divergent transcriptomic identities of cell types that share a common neurotransmitter (Fig. 1C).

In telencephalic structures such as neocortex, hippocampus, and amygdala, glutamatergic neurons express *SLC17A7* (VGLUT1) and segregate from GABAergic telencephalic neurons (Fig. 1C). Yet, even within the telencephalon, neurotransmitter identity did not drive global transcriptional similarity between major clades. GABAergic projection neurons, such as spiny projection neurons (SPNs) of the striatum, were transcriptionally closer to telencephalic glutamatergic neurons than SPNs were to telencephalic GABAergic interneurons (Fig. 1C).

Although glutamatergic neurons from distinct cephalic origins do not cluster together, maintaining glutamatergic neurotransmission or associated function could require a common core set of genes. To assess how neurotransmitter utilization relates to genome-wide RNA expression patterns, we examined distributions of gene-gene correlations across cell types (Fig. 1F). Surprisingly few genes are strongly positively correlated with both *SLC17A6* (VGLUT2) and *SLC17A7* (VGLUT1) expression, even those associated with glutamate synthesis and packaging. One hundred sixteen genes had correlated expression to *SLC17A7* (Spearman’s tau > 0.5). The median correlation of *SLC17A6* to those 116 genes was centered at tau = 0.05 (Fig. 1G). Only a few genes correlated above tau = 0.5 to both *SLC17A6* and *SLC17A7,* including *BDNF, NRN1*, and *TAFA1.* Moreover, only 10 genes (*ARPP21*, *BDNF*, *CACNA2D1*, *CHN1*, *CHST8*, *CPNE4*, *LDB2*, *NRN1*, *PTPRK*, and *TAFA1*) are differentially expressed (>2.5-fold change) in both telencephalic (*SLC17A7*^+^) glutamatergic neurons and nontelencephalic (*SLC17A6*^+^*, SLC17A7^−^*) glutamatergic neurons relative to GABAergic (*GAD1*^+^) neurons. The bulk of gene expression that distinguishes neuronal types from each other appears incidental to their neurotransmitter identities.

### Adult neuronal types are imprinted by their developmental origin

**Fig. 2.**
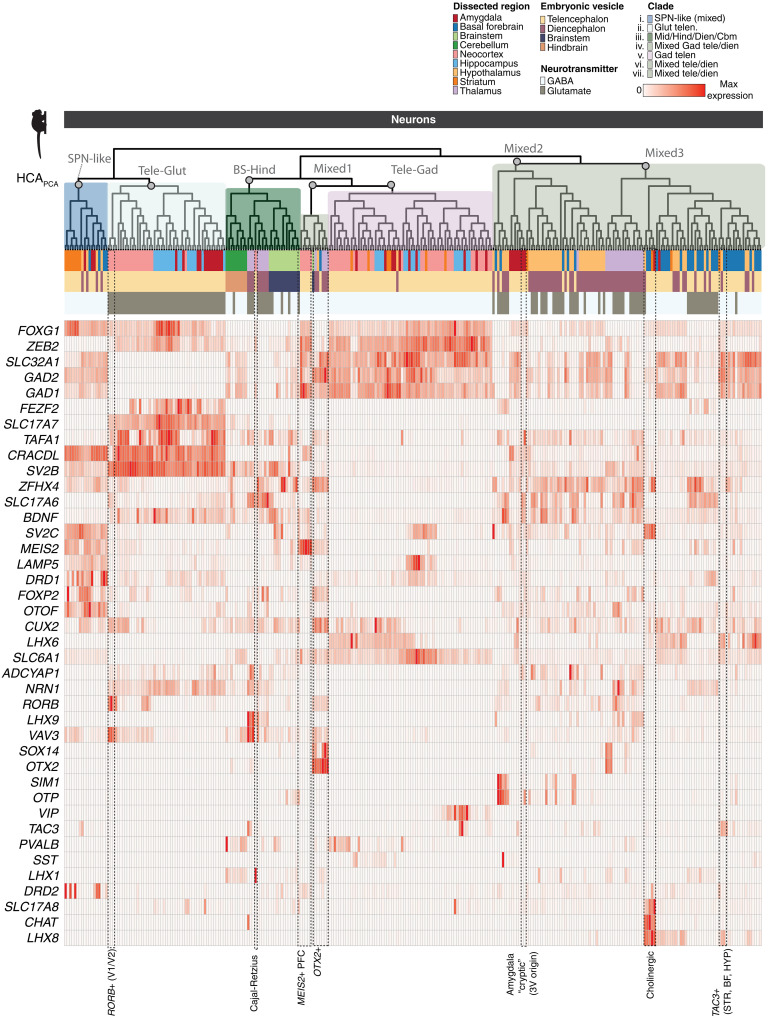
Gene expression across neural populations. Expression of broad class marker genes and other genes of interest across all neurons sampled by snRNA-seq. Heatmap colors are scaled to max normalized expression for each row (gene). Dendrogram ordering and metadata colors as in Fig. 1C. Cell types discussed in the main text are labeled at the bottom.

We inspected RNA expression patterns along with details about each cell type’s dissection region of origin to assess which brain structures tended to contain highly similar cell types and which had more dispersed transcriptional profiles. Some tissues, such as the neocortex, gave rise to cell types that exclusively clustered within their cephalic domain (Fig. 1C), a result unlikely to be driven by ambient RNA contamination since regionally variable genes were not over-enriched in neocortical ambient RNA estimates (fig. S3A). However, within a given cephalic domain, cell types from distinct brain structures were often more similar to types sampled from other brain structures. For example, while hippocampal cell types were all found in telencephalic clades in the dendrogram, many individual hippocampal types were more like types in the amygdala or neocortex than they were like other hippocampal types (Fig. 2).

Out of 62 neocortical neuron types, only two types joined clades outside of the major GABAergic and glutamatergic telencephalic branches: (i) the *MEIS2*^+^ prefrontal GABAergic types (fig. S1C and Fig. 2), which formed a clade most similar to diencephalic and midbrain *OTX2*^+^ types, and (ii) neocortical Cajal-Retzius neurons, which were more similar to a clade of *LHX9*^+^ thalamic neurons despite indications that they predominantly originate from the cortical hem in primates (Figs. 1E and 2) (*40*). Telencephalic interneuron subtypes tended to cluster with those that express the same class marker. For example, all *PVALB*^+^ types sampled across neocortex, amygdala, and striatum clustered closely together (Fig. 2), consistent with a shared developmental origin. 

Consistent with previous work which suggested that mammalian thalamus contains both midbrain-derived and forebrain-derived GABAergic interneurons (*41*), we observed distinct clades of thalamic neurons that were most similar to diencephalic or midbrain populations. Thalamic GABAergic neurons that were *OTX2*^+^ were distinct from other thalamic populations (Figs. 1E and 2) and formed a clade with other *OTX2*^+^ neurons sampled from brainstem, hypothalamus, and basal forebrain. These populations were *SOX14*^+^, while thalamic *SOX14*^−^ populations joined mixed diencephalic-telencephalic clades (Figs. 1E and 2). The dispersion of thalamic neurons to distinct diencephalic and midbrain-dominated clades supports recent work suggesting multiple developmental origins for primate thalamic GABAergic neurons (*41*). We did not find thalamic GABAergic populations that were transcriptionally similar to telencephalic types (*42*), potentially owing to nonexhaustive sampling of thalamus, although we note that the next most proximal clade to the thalamic *OTX2*^+^ types contained *MEIS2*^+^ GABAergic neocortical neurons (Fig. 2).

The amygdala is composed of loosely associated nuclei with diverse phylogenetic and developmental origins (*43*, *44*), and amygdala neuron types distributed widely across the dendrogram (Fig. 1C). Amygdala excitatory neurons, which share properties with cortical and claustrum neurons, clustered with other excitatory telencephalic neurons. In contrast, *FOXP2*^+^ amygdala GABAergic neurons, which reside in the intercalated nuclei (*19*, *45*, *46*), cluster with striatal SPNs (Fig. 2), in line with recent lineage tracing studies in mice (*19*) and with analysis of fetal macaque cell types (*46*). These results underscore the diversity and developmental complexity of cell types comprising the amygdala nuclei.

### Brain cell taxonomies are conserved and are shaped by broad classes of genes

**Fig. 3.**
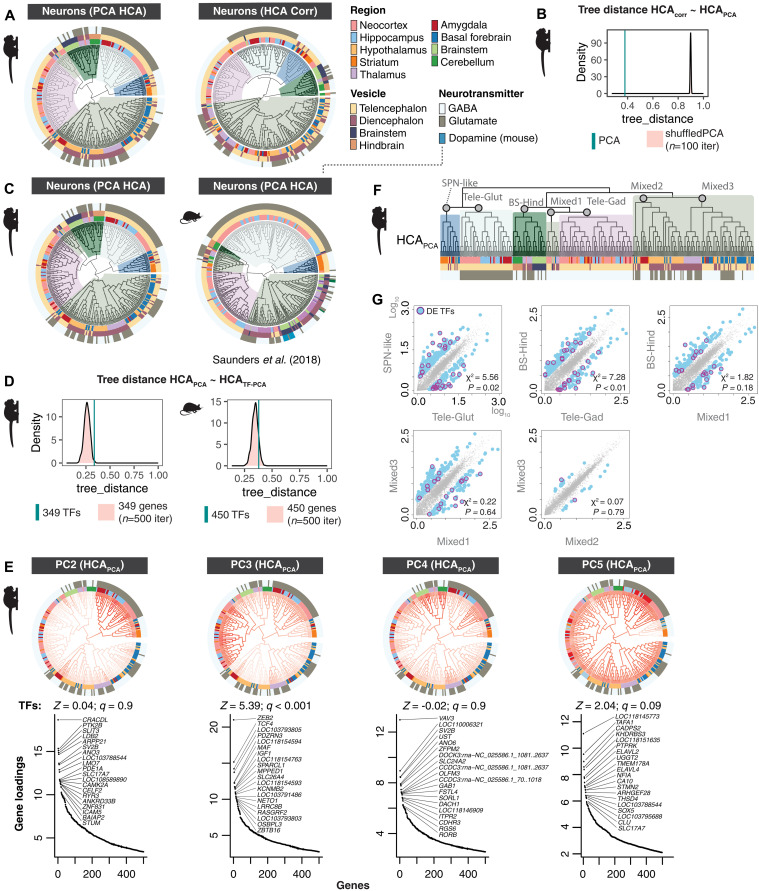
Conservation of neuronal hierarchy across species and clustering methods. (**A**) Comparison between hierarchical clustering (HCA) of marmoset neurons computed on top 100 principal components (PCA) or from a correlation-based distance measure (HCA Corr). (**B**) Distance between trees computed in (A). Cyan line = tree distance between HCA PCA and HCA Corr. Pink distribution is tree distance scores between HCA_corr_ and shuffled PCA scores (*n* = 100 shuffling iterations). Lower values of tree_distance (*x* axis) indicate higher agreement between dendrogram tree structures. (**C**) Comparison of marmoset and mouse neurons [mouse atlas data from (*4*)] using HCA PCA on 5884 genes in marmoset and 3528 1:1 orthologs in mouse. (**D**) Tree distance comparisons to shuffled distributions using expressed TFs in marmoset and mouse neurons. (**E**) Top gene loadings for PC2 to PC5 computed over marmoset metacells (table S2). PC scores are plotted on the HCA PCA dendrogram shown in Figs. 1C and 2. Ranked gene loading plots show top 20 genes per PC. *Z* scores and *q* values show false discovery rate (FDR)–corrected tests of TF enrichments in top loading genes per PC. (**F**) Marmoset dendrogram in Fig. 1C (HCA_PCA_) indicating major clades compared in (G). (**G**) “Ancestral” reconstruction (AR) of gene expression profiles of major clades of marmoset neuron types from HCA PCA dendrogram using maximum likelihood estimates. Scatterplots show pairwise comparisons between AR of internal nodes of major clades. Blue dots, genes with >3-fold change difference between the two ARs; magenta circles, differentially expressed TFs (DE-TFs). Chi-square and *P* values describe whether TFs are significantly differentially enriched between the AR pairs.

We assessed whether the transcriptomic similarities observed using our hierarchical clustering approach (HCA-PCA) held if using different methods or datasets (Fig. 3). First, we found that the overall dendrogram configuration was broadly conserved when marmoset neuron hierarchical clustering was recomputed using other distance functions (Fig. 3A). To determine whether a consistent pattern of transcriptomic similarity is found in other species, we next repeated PCA-based HCA clustering on a publicly available single-cell RNA sequencing atlas of the adult mouse brain (*4*).

We found a similar global tree structure between the marmoset and mouse neurons, suggesting broad conservation of the features that drive transcriptomic similarities in neurons across adult primates and rodents (Fig. 3, B and C). In particular, the relative ordering of the major clades was strikingly similar across species: For example, in both species, telencephalic glutamatergic neurons are most similar to a clade of GABAergic neurons that includes striatal SPNs as well as transcriptionally similar types of GABAergic, *FOXP2*^+^ neurons in amygdala and hypothalamus (Fig. 3, B to D). Other examples of concordance between species included that all telencephalic GABAergic interneurons formed a large single clade, and the presence of a mixed clade containing thalamic and brainstem types. Cerebellar neuron types were entirely restricted to a single, unmixed clade in both mouse and marmoset (Fig. 3B), although this relative isolation could be due to the lack of other hindbrain structures in the datasets.

Transcription factors (TFs) are master regulators that determine cell type identity in development through temporal patterning, suggesting that they may constitute a key class of genes that maintain transcriptional identity in adults (*1*). Supporting this view, some TFs are strongly associated with specific brain structures or cephalic compartments, such as *FOXG1* in the telencephalon (Fig. 1E) and *OTX2* in nontelencephalic structures (Fig. 1E). Hierarchical clustering based only on TF genes (349 genes in marmoset, 450 in mouse) was highly similar to the neuronal trees that were computed over all expressed genes (5882 in marmoset, 3528 homologous genes in mouse) (Fig. 3C). However, tree ordering generated by TFs alone did not produce lower tree distances to the original tree compared to similarly sized sets of randomly selected expressed genes (Fig. 3D). This suggests that although TFs undoubtedly play a central role in determining cell identity in development, the suite expressed in adult types are not unique in reflecting the transcriptomic identities of adult neuron types as a whole. Out of the top 20 PCs (which account for 92% variance), 7 PCs were enriched for TFs within the top 200 genes (absolute loadings) compared with randomly sampled genes. Several individual PCs (table S2) loaded on specific clades of the tree, but most showed more complex patterns across clades (Fig. 3E), reinforcing that adult TF expression captures aspects of transcriptional identities but does not drive global transcriptional similarity across neurons.

Borrowing from ancestral state reconstruction methods typically used to estimate evolutionary divergences between genetic sequences or species (*47*), we applied a maximum likelihood–based approach (fastAnc) to the expression of all genes at each leaf of the marmoset dendrogram (corresponding to observed metacells), along with the branch lengths between adjacent leaves, to reconstruct the transcriptomic state (inferred expression values) at internal nodes of the dendrogram (Fig. 3, F and G). We then compared pairwise “ancestral” expression values of all genes in the parent nodes for each of the seven major clades depicted in Figs. 1C and 3 (F and G). TFs were overrepresented in comparisons of internal nodes that contained cell types stemming from developmentally related brain regions, but were not overrepresented when the leaf clusters stemmed from multiple regions (Fig. 3, F and G). While the expression of some TFs reflects the developmental origins of neurons, some cell types are transcriptionally similar despite having distinct developmental origins. This may reflect aspects of convergence in adult transcriptional profiles (*8*, *19*).

### A hypothalamic-origin neuron type in medial amygdala retains its diencephalic identity

In mammalian CNS, some cell types migrate long distances from proliferative zones to their mature destinations (*48*, *49*). However, neurons generally tend to respect cephalic boundaries and remain within the same subdivision as their progenitors (*50*). This tight control over migration potential makes it difficult to disentangle the persistent influence of developmental origin from later influences arising from shared tissue context, which could affect all neurons similarly within a brain structure. Cephalic boundary crossings, though rare, do exist (*41*, *42*, *51*). Such boundary crossing events can reveal whether cell types that embarked on cross-cephalic migration retain transcriptomic profiles more in common with their tissues of origin, or more in common with their final destinations.

**Fig. 4.**
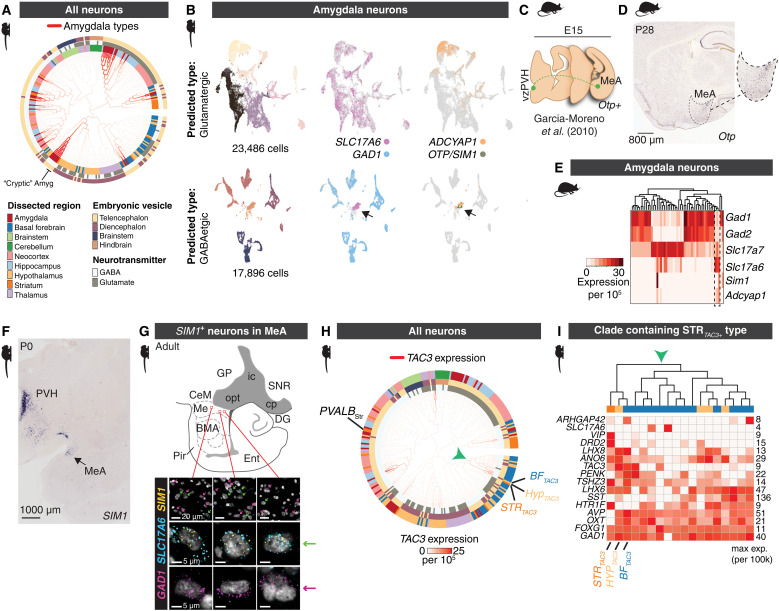
Examples suggesting cross–cephalic boundary cell type migration. (**A**) Locations of amygdala clusters in the neuronal HCA-PCA dendrogram from Fig. 1C. (**B**) Clustering of marmoset amygdala cells (n = 44,165 neurons) predicted to be glutamatergic (top row) or GABAergic (bottom row) based on a linear classifier (scPred) trained on supervised cell type models. t-distributed Stochastic Neighbor Embedding (t-SNEs) for each class are colored by cluster or positive expression of genes (*SLC17A6*, *GAD1, ADCYAP1, OTP, SIM1*). Arrowhead indicates cluster of neurons that are classified as GABAergic, yet do not express *GAD1* and do express *SLC17A6*. (**C**) Cartoon of embryonic migration of *Otp*^+^ cells that migrate from proliferative zones around the third ventricle to periventricular hypothalamus (vzPZH) and medial amygdala (MeA), following data in (*51*). (**D**) ISH for *Otp* in sagittal section of P28 mouse brain. Dotted outline indicates borders of medial amygdala (MeA). (**E**) snRNA-seq from mouse amygdala neurons. Heatmap shows normalized, scaled expression (n = 25,930 nuclei, 3 replicates pooled). Dendrogram shows hierarchical clustering of neuron types based on all expressed genes. Dotted outline shows the presence of three Slc17a6+ subtypes that preferentially cluster with GABAergic (GAD1+/GAD2+) subtypes and that express Sim1 and/or Adcyap1. (**F**) Marmoset P0 coronal section showing ISH staining for *SIM1* from the marmoset BRAIN/Minds atlas (*59*, *60*). (**G**) Cartoon of amygdala in sagittal plane of adult marmoset. FISH staining for *GAD1* (magenta), *SLC17A6* (cyan), and *SIM1* (yellow) in the medial amygdala (Me). Magenta arrows highlight *GAD1*-expressing nuclei. Green arrows highlight *SLC17A6* and *SIM1* coexpression. Scale bars, 20 μm (top row) and 5 μm (middle and bottom rows). (**H**) Marmoset neuronal HCA PCA dendrogram with branches colored by *TAC3* expression. Unexpectedly, a primate-specific striatal *TAC3* population (*10*) clusters with hypothalamic and basal forebrain populations. Arrowhead indicates clade containing *TAC3*^+^ striatal type. (**I**) Heatmap of genes expressed in the clade indicated by green arrowhead in (H). Expression values of each gene are normalized to its max within the clade.

Our data confirm a striking example of cross-cephalic migration in the amygdala. First, we observed an unexpected clustering pattern in our analysis: Despite expressing *SLC17A6* and lacking expression of *GAD1* and *GAD2,* three amygdala neuron clusters showed a closer association with GABAergic rather than glutamatergic types (Fig. 4, A and B). They did not express other genes required for GABAergic transmission such as *SLC32A1* (VGAT) and also lacked the molecular machinery for noncanonical GABA reuptake or release that has been observed in other cell populations (Fig. 2) (*52*, *53*). These findings indicate that these particular amygdalar neurons exhibit a “cryptic” transcriptomic identity, in which they are presumably glutamatergic yet have global transcriptional profiles that are more similar to GABAergic types, relative to other telencephalic neurons.

The cryptic amygdalar subtypes display additional atypical gene expression features compared to other amygdala neuronal types, including expression of *OTP* and *SIM1* (Fig. 4B). These TFs are typically expressed in neuronal lineages originating from proliferative zones around the third ventricle. Previous work from other laboratories has shown that in mice, a migratory stream of cells labeled from third ventricle electroporation crosses into the telencephalon (*51*, *54*) and reaches the medial amygdala. This migration is dependent on *Otp* expression; subtypes also express *Sim1* (Fig. 4C) (*51*). To establish whether these cells are detectable in mouse amygdala, where they were initially described (*51*, *54*), we generated an snRNA-seq dataset consisting of 53,745 nuclei from adult mouse amygdala (P60 to P70). Otp protein expression in mouse amygdala becomes largely down-regulated in adulthood (*51*), although *Otp* mRNA is present in the medial amygdala in P28 mice (Fig. 4D). Consistent with down-regulation of Otp protein over development, we did not detect *Otp* gene expression in our snRNA-seq dataset; however, we detected several neuronal clusters that expressed *Sim1* (Fig. 4E).

Mirroring the cryptic transcriptomic profile we observed in the *OTP*^+^*/SIM1*^+^ marmoset amygdala neuron types (Fig. 4, A and B), the mouse amygdala *Sim1*^+^ neurons clustered with GABAergic neurons, yet expressed *Slc17a6* but not *Gad1* or *Gad2* (Fig. 4E). In mice, these neurons also constitute the majority population in the amygdala that express *Adcyap1* (encoding the protein PACAP) (Fig. 4E), a neuropeptide that is extensively (but not exclusively) expressed in hypothalamic populations (*55*) and associated with energy homeostasis (*56*), stress, anxiety (*57*), and immune responses (*58*). [In marmosets, *ADCYAP1* is additionally expressed in subsets of *SLC17A7*^+^ neurons that do not share the cryptic phenotype (Fig. 4B), indicating that *ADCYAP1* has a distinct pattern of expression in amygdala neurons between mice and marmosets.]

To precisely locate the specific amygdalar nuclei housing the cryptic population, we investigated the expression of *SIM1* in marmosets (*59*, *60*). *SIM1* expression was highly enriched in the neonatal marmoset medial amygdala (MeA) (https://gene-atlas.brainminds.jp; Fig. 4F), which mirrors the migration of *Sim1/Otp*^+^ neurons from the diencephalon in mice into medial amygdala (*51*). We next verified *SIM1* and *SLC17A6* but not *SIM1* and *GAD1* colocalization in adult marmoset medial amygdala using smFISH (Fig. 4G).

On the basis of these observations, *SIM1*^+^*/OTP*^+^ amygdala neurons likely cross from the diencephalon into the telencephalon early in development in both species. This offers a test of the persistence of developmental origin on transcriptomic identities: If amygdalar *SIM1*^+^*/OTP*^+^ neurons are globally more similar to hypothalamic types, it suggests that they retain developmental imprint of their origin despite crossing into the telencephalon by P0 (Fig. 4F). We inspected our marmoset neuronal dendrogram to determine whether these amygdala-resident types preferentially associated with telencephalic neurons or diencephalic types. The cryptic amygdala neuron types (as well as several other small amygdala populations that are not *SIM1*^+^ or *OTP*^+^ but that are transcriptionally similar to the amygdala cryptic types) join a clade with hypothalamic and basal forebrain types (Fig. 4A), separate from most other amygdala types that reside in telencephalic clades. These results suggest that the cryptic amygdala neurons are a conserved population in both mice and primates that likely have diencephalic origins. Consistent with a birthplace imprinting effect, they retain transcriptional identities more similar to diencephalic types than to telencephalic types with which they ultimately reside, despite their early migration into the telencephalon (Fig. 4A).

### Primate-specific striatal TAC3^+^ interneurons are similar to specific TAC3^+^ diencephalic types

Previously, we discovered a *TAC3^+^, LHX6^+^* interneuron subtype in the striatum of humans, macaques, and marmosets that was absent in mice and ferrets (*10*). Compared with other striatal types, they are transcriptionally most similar to *PVALB^+^* interneurons and, because they expressed the TF *LHX6*, we surmised that they likely also arose from the medial ganglionic eminences (MGEs) (*10*). Supporting this inference, a recent study of fetal macaque snRNA-seq data from ventral telencephalic progenitor domains found that primate *TAC3^+^* striatal interneurons likely arise from progenitors in the MGE, which diverge from an ancestral progenitor class that also gives rise to conserved *MAF^+^* progenitors that go on to produce *PVALB^+^* and *TH^+^* striatal interneurons (*46*).

The broader census of brain structures in the current dataset allowed us to examine the transcriptional identity of the striatal *TAC3^+^* type relative to cell types outside of the striatum. *TAC3* is expressed in 20 different neuron types in our dataset, including in cortical GABAergic neurons as well as several amygdala, basal forebrain, thalamic, and hippocampal types (fig. S4, A to C, and Fig. 2). *TAC3^+^* types did not form a single clade in the dendrogram, and their transcriptional resemblance to other neuron types largely reflected their tissue or cephalic origin. For example, thalamic *TAC3^+^* subtypes were found in a thalamic-only clade, while hippocampal *TAC3^+^* neurons were most similar to other hippocampal and amygdala types. The thalamic types were *SLC17A6^+^*, while all other *TAC3^+^* types were GABAergic. These results suggest that *TAC3* expression alone does not mark a transcriptionally uniform set of cell types.

Unexpectedly, the striatal *TAC3^+^* type was most similar not to other striatal interneuron types as we previously concluded (*10*), but rather to two other *TAC3^+^* GABAergic types found in basal forebrain and hypothalamus (Fig. 4H, *TAC3* smFISH in fig. S4, A and B, and data S1). The broader clade containing these three types (depicted by green arrowhead in Fig. 4I) otherwise consisted entirely of basal forebrain and hypothalamic types. Each of the three *TAC3^+^* populations had distinct gene expression (such as high expression of *OXT* and *AVP* in the hypothalamic type, and *DRD2* expression exclusively in the striatal type), ruling out dissection contamination (Fig. 4I). The three types remained direct neighbors when the dendrogram was recomputed using other distance metrics (e.g., correlation-based or PCA scores using TF expression; see Fig. 3).

As the similarity of the *TAC3^+^* striatal type to hypothalamic and basal forebrain neurons was unexpected, we omitted the two basal forebrain and hypothalamic *TAC3^+^* metacells and recomputed the dendrogram using HCA PCA (retaining 286 of the original 288 neuronal types) to determine whether *TAC3^+^* striatal types were globally most similar to hypothalamic neurons (fig. S4C). ​​The global structure of the dendrogram was essentially the same as the original, except that the striatal *TAC3^+^* type joined the major telencephalic GABAergic clade with other striatal interneurons (neighboring the striatal *PVALB^+^* subtype) (fig. S4C), recapitulating our original assessment that considered their similarity only to other striatal interneurons (*10*). This suggests that TAC3*^+^* striatal types are not globally most similar to hypothalamic resident neurons, but that they share a particularly close identity to a specific subset of hypothalamic and basal forebrain types that also express *TAC3.*

Considering their unexpected transcriptional similarity to both a telencephalic (basal forebrain) and a diencephalic (hypothalamus) type, the *TAC3^+^* striatal type may arise from a telencephalic progenitor (*46*) that also gives rise to sister diencephalic (hypothalamic) types, or else shows striking transcriptional convergence with diencephalic types that have distinct developmental origins. Favoring a telencephalic origin as suggested by previous report (*46*), the hypothalamic, basal forebrain, and striatal *TAC3^+^* types are all *FOXG1^+^*, a TF associated with telencephalic origin (Fig. 4I). They also express *LHX6^+^* and *NKX2-1^+^* (Fig. 2), consistent with an MGE origin; however, we note that in mice some hypothalamic types also express *Nkx2-1* and *Lhx6* (*30*, *61*, *62*). Ultimately, lineage tracing of the striatal *TAC3^+^* type in a primate would resolve whether a shared progenitor gives rise to both telencephalic and diencephalic types.

### Neocortical expression fingerprints differ between neurons and glia

Our cross-structure analysis suggests that in the marmoset brain, global neuronal taxonomies are influenced by broad developmental factors such as cephalic origin (Figs. 1 to 4). However, there is also considerable within-structure heterogeneity of cell types and proportions, particularly in highly patterned structures like the neocortex. We previously showed that primate cortical interneurons regionally varied in their gene expression considerably more than mice, suggesting an evolutionary distinction (*10*). Within the human neocortex, molecular divergence across functionally specialized areas is already present in cortical progenitors, suggesting that early developmental processes shape intra-cortical cell identities (*63*, *64*). However, it is unclear whether intra-cortical regional changes are shared or distinct across distinct cell classes such as neurons and glia.

**Fig. 5.**
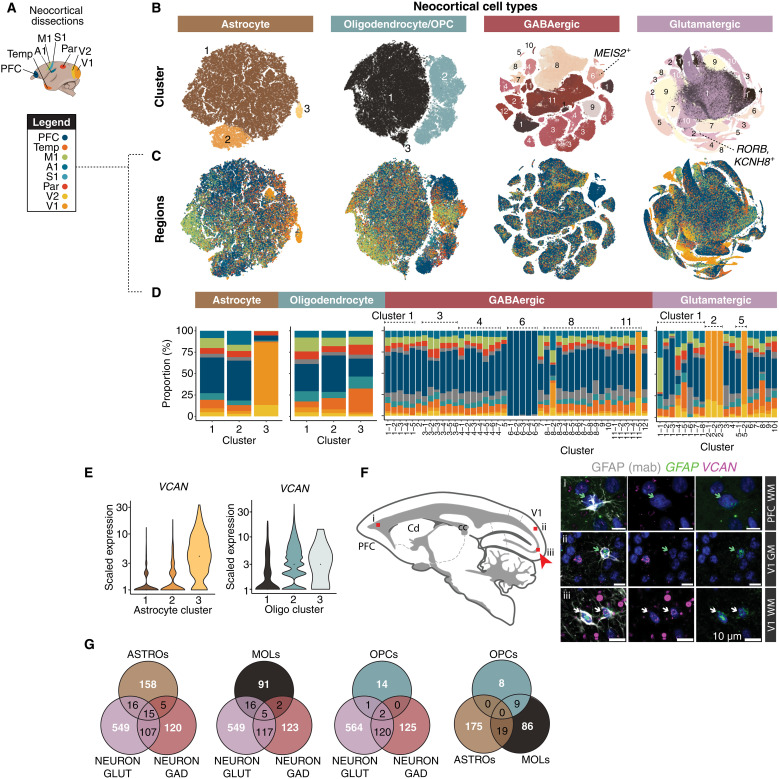
Regional variation in neocortical cell types and expression patterns. (**A**) Cortical regions sampled. (**B**) t-SNE embeddings of neocortical macroglia (astrocytes, oligodendrocyte lineage types) and neurons (GABAergic interneurons, glutamatergic neurons). Colors represent clusters (numbered). (**C**) Same as (B) but cells colored by neocortical dissection. (**D**) Regional proportions of each cluster; colors same as (A). (**E**) *VCAN* expression across astrocyte clusters and oligodendrocyte lineage clusters. Colors as in (B). (**F**) (Left) Cartoon of sagittal section imaged; red boxes (i to iii) correspond to (right) tissue validation of increased abundance of *VCAN*^+^ astrocytes in adult marmoset V1-adjacent white matter (iii) compared with PFC-adjacent white matter (i) and V1 gray matter (ii). GFAP antibody (gray) combined with smFISH probes against *VCAN* (magenta) and *GFAP* (green). Green arrows correspond to *GFAP*^+^ (antibody) and *GFAP*^+^ (smFISH) cells. White arrows correspond to GFAP^+^ (antibody), *GFAP*^+^ (smFISH), and *VCAN*^+^ cells. V1, visual cortex V1; PFC, prefrontal cortex; GM, gray matter; WM, white matter. Red arrow highlights locale of *VCAN*^+^ *GFAP*^+^ cells. Scale bar, 10 μm. (**G**) Venn diagrams showing overlap of neocortical regionally differentially expressed genes (rDEGs) across GABAergic neurons, glutamatergic neurons, astrocytes, and oligodendrocyte lineage cells (MOLs and OPCs). rDEGs are defined as >3-fold expression difference in homologous cell types across pairs of cortical regions.

We examined regional distinctions in proportions and gene expression of neocortical cell types from eight locations (Fig. 5A). Consistent with prior studies across a range of mammalian species (*4*, *10*, *20*, *65*), cell subtypes identified in one cortical region were generally present in all other cortical regions, though in different proportions (fig. S1B). There were two main exceptions in neurons. GABAergic *MEIS2^+^* cells (GABAergic cluster 6; Fig. 5B) were far overrepresented in PFC samples (particularly in dissections of medial and medio-orbital PFC; fig. S1C), a compositional distinction that is not observed in mouse (*4*, *65*), and that may reflect the differential routing of MEIS2*^+^* neurons into primate neocortex instead of olfactory bulb as they do in mice (*46*). The second exception was a cluster of *RORB^+^, KCNH8^+^* glutamatergic neurons in V1 (and to a lesser extent V2) that was distinct from *RORB^+^* populations found in the other cortical regions (glutamatergic cluster 2; Fig. 5B). The expansion and divergence of *RORB^+^* populations in visual cortex is consistent with the elaboration and subspecialization of primary visual cortex layer IV in primates (*66*).

Glutamatergic neurons sampled from different neocortical locations show regionally distinct gene expression (*65*). In primates, this is also true of neocortical GABAergic neurons (*10*). However, the extent to which glial cells are locally customized in distinct regions of neocortex is less well understood (*5*, *8*, *17*, *18*, *67*). Studies in mice have revealed layer-specific astrocyte subpopulations in the cortex (*68*) as well as variation between neocortical and hippocampal astrocytes (*69*). We found that one astrocyte type, marked by high expression of *VCAN*, was highly enriched in marmoset occipital lobe dissections (V1 and V2) (astrocyte cluster 3; Fig. 5B). *VCAN* is also expressed in OPCs, but we did not observe higher *VCAN* expression in V1 OPCs (Fig. 5E). Using smFISH, we validated higher colocalization of *VCAN* and *GFAP* in V1-adjacent white matter compared with PFC white matter and V1 gray matter (Fig. 5F), suggesting that within the neocortex, the regional variation in *VCAN* expression is specific to white matter–resident occipital astrocytes.

To address whether cortical regional variation in gene expression is shared across cell types, we performed pairwise comparisons between major clusters of cortical excitatory neurons, inhibitory neurons, astrocytes, and oligodendrocyte lineage types across all eight neocortical locations (Fig. 5E and table S3). Because primate cell types show considerable interindividual variability in gene expression from genetic and other sources (*6*, *70*), we calculated regionally differentially expressed genes (rDEGs) for each of the three donors for which we had sampled the complete set of eight cortical locations, and took the consensus set of rDEGs across individuals. Each cell class displayed rDEGs across neocortical regions (Fig. 5G), an effect that could not be attributed to ambient RNA contamination (fig. S3A). Similar to what has been described in the mouse cortex (*68*, *69*), astrocytes within the marmoset cortex exhibited considerable regional transcriptional variation, but overall neurons had more rDEGs than macroglia (Fig. 5G and fig. S3B). Forty-nine percent of rDEGs in interneurons were also rDEGs in excitatory neurons (Fig. 5G). Although cortical astrocytes and oligodendrocyte lineage cells arise from a closer common lineage with cortical excitatory neurons [dorsal radial glia (*71*)], they shared a lower percentage of rDEGs in common with excitatory neurons (16% astrocytes, 18% mature oligodendrocytes, 17% OPCs) than the fraction of rDEGs that were shared between excitatory neurons and interneurons, which arise from distinct progenitor populations (*72*) [though see (*73*, *74*)].

rDEGs within a cell class (glutamatergic, GABAergic, astrocyte, oligodendrocyte lineage) tend to be biased in the same regions across subtypes within that class. However, certain subtypes and regions accumulated more rDEGs than others. For example, across all cell types and particularly within neurons, higher-order temporal association cortex and PFC tended to be most distinct from V1 and V2 (fig. S3B). There was also cell type variability in the extent to which rDEGs were private to individual donors (table S3): 55% and 39% of glutamatergic and GABAergic rDEGs, respectively, were shared among donors, compared to 24% and 14% shared astrocyte and oligodendrocyte rDEGs. These results may indicate lower interindividual consistency in regional gene expression in marmoset nonneuronal cells, although larger sample sizes are needed to determine underlying genetic, environmental, or technical sources (*6*, *70*).

### Interneurons distribute distinctly across primate neocortex and striatum

Our analysis of RNA expression from the single-nucleus dataset indicates that developmentally linked telencephalic GABAergic populations (*48*, *49*) retain globally similar identities in adulthood. For example, *SST^+^* striatal interneurons are more similar to *SST^+^* hippocampal and neocortical interneurons than they are to other striatal interneuron types (Fig. 2). Within each brain structure, developmentally linked populations become functionally specialized and follow distinct spatial rules for their allocation. In the mouse, quantitative imaging of molecularly-identified interneuron types has revealed that densities of *Sst^+^*, *Vip^+^*, and *Pvalb^+^* cortical interneurons vary across the cortical mantle; their relative proportions relate to unique functional and microcircuit properties of different cortical areas (*22*). In primates, overall neuron densities are more spatially variable than in mice: They vary by as much as fivefold across the cortical sheet, with highest neuron proportions and densities found in occipital cortex and particularly in V1 (*75*). However, quantitative mapping of molecularly-defined subtypes of neurons across areas has not been performed in a primate, and it is not known if they have conserved or distinct spatial distributions compared to mice.

**Fig. 6.**
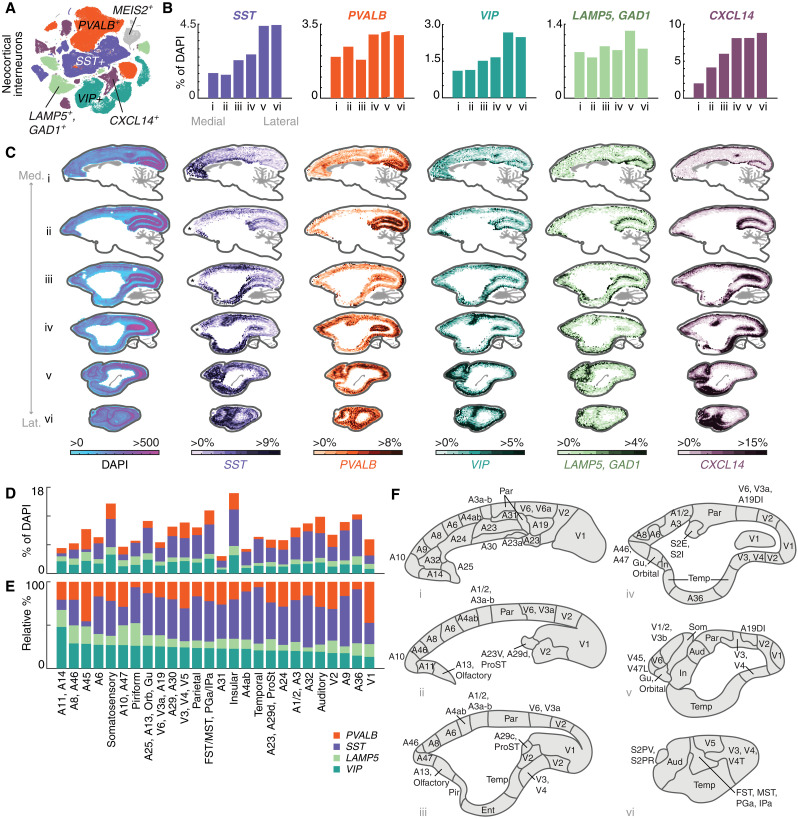
Cell type–specific distributions of interneurons in marmoset neocortex using quantitative smFISH. (**A**) t-SNE of GABAergic neocortical interneurons, colored by subclass marker [*PVALB, SST, VIP, LAMP5* (*GAD1*^+^)*, CXCL14*]. Gray points are the *MEIS2*^+^ population that is restricted to orbitomedial PFC (fig. S1, B and C) and was not spatially profiled. (**B**) Medial-lateral proportions of each major class as percentage of all cells (DAPI^+^). Barplots quantify positive cells as proportion of all (DAPI^+^) cells from medial to lateral sections of smFISH performed on six thin (16 μm) sagittal sections of marmoset neocortex, each section being 1600 μm apart and covering 9600 μm of neocortex. Colors as in (A). (**C**) smFISH for neocortical interneuron subclass markers showing locations of cells positive for each marker across six sagittal sections of the marmoset neocortex. First column shows density of all DAPI^+^ nuclei per unit area (approximately 387 μm per bin) profiled from one series. Heatmap scale in subsequent columns shows percentage of marker-positive cells relative to DAPI^+^ cells. Average proportions across section shown in (B). Med., medial; Lat., lateral. Asterisks in (ii) and (iii) of the *SST* series and (iv) of the *LAMP5* series denote regions where tissue or staining artifacts caused loss of signal. These can be seen in the DAPI-only series of these experiments, which show lower overall cell counts at these locations (fig. S5). (**D**) Quantitation of interneuron proportions by cortical area parcellated according to (F). (**E**) Relative percentages of interneuron proportions by cortical area shown in (F), sorted by max relative proportion of *VIP*^+^ interneurons. (**F**) Cartoons of cortical areas and areal groupings used to bin smFISH neocortical interneuron proportions from (A) in (D) and (E). Neocortical parcellation from (*76*).

**Fig. 7.**
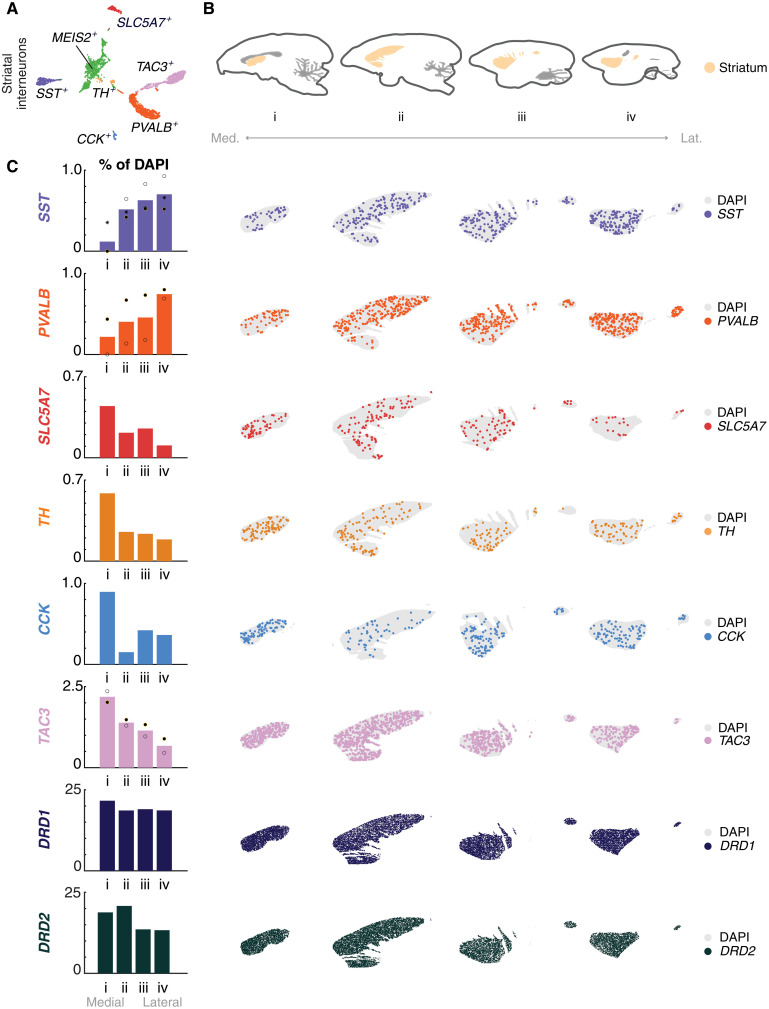
Cell type–specific distributions of interneurons in marmoset striatum using quantitative smFISH. (**A**) t-SNE of striatal, cholinergic neurons (*SLC5A7*) and GABAergic interneurons (*SST*, *PVALB*, *TH*, *CCK*, *TAC3*). Green points correspond to the *MEIS2*^+^ population and were not spatially profiled. (**B**) Cartoon of marmoset striatum illustrating the area profiled, medial to lateral. (**C**) (Left) Medial-lateral gradients of striatal cell type proportions across four sagittal sections. Dots, individual replicates. Colors as in (A). *DRD1*, dark blue; *DRD2*, dark green. (Right) Scatterplot of smFISH for striatal cholinergic and interneuron subclass markers, as well as dopaminergic striatal populations [*DRD1*^+^ (dark blue) and *DRD2*^+^ (dark green)], showing locations of cells positive for each subclass marker across four (16 μm) sagittal sections of the marmoset striatum, each 1600 μm apart covering 6400 μm in total.

We used smFISH to image the distributions of major cortical and striatal interneuron types across sagittal sections in marmoset (Figs. 6 and 7; figs. S5 to S7). We quantified proportions and densities of each type relative to all cells [4′,6-diamidino-2-phenylindole (DAPI); fig. S5]. In the neocortex, we binned these using an areal parcellation of marmoset neocortex (*76*) to determine whether interneuron proportions varied systematically by brain area. In total, we quantified 377,554 neocortical interneurons across 30 sections by smFISH from one marmoset donor (Cj 18-134; table S1). In striatum, we quantified 6848 interneurons across 32 sections. Each series sampled sagittal sections ~160 μm apart, beginning 1184 to 1584 μm from the midline up to 6384 μm laterally, including most of the striatum with the exception of the lateral-most portion of the putamen. We used a cell segmentation algorithm to count positive cells across sagittal sections and expressed interneuron proportions as a percent of all cells (DAPI*^+^*).

Neocortical types were identified with probes for *SST*, *PVALB*, *CXCL14*, *VIP*, and *LAMP5* (table S4), which collectively account for all major cortical interneuron populations (Fig. 6 and figs. S5 and S6). *CXCL14* is a marker for caudal ganglionic eminence (CGE)–derived cortical interneurons. It is expressed in most *VIP^+^* and *LAMP5^+^* cortical neurons, as well as in a smaller population of *VIP*^−^, *LAMP5*^−^ types, some of which are *PAX6^+^* [and which correspond to the *SNCG^+^* population in humans and mice (*10*, *17*)]. As *LAMP5* is also expressed in subsets of excitatory neurons, we performed dual-labeling smFISH with *GAD1* to avoid counting glutamatergic types. Major striatal interneuron types were identified with probes for *SST, PVALB, CHAT/SLC5A7, TH, CCK,* and *TAC3* (table S4). These together account for most major populations of non-SPN neurons in the striatum, with the exception of a population of *MEIS2^+^* GABAergic striatal neurons that cluster together with non-SPN GABAergic interneurons (Figs. 1C and 2) but are difficult to distinguish uniquely using smFISH, since several other markers are also expressed at variable levels in other striatal cell types.

### Neocortical interneuron types have highly focal biodistributions

Quantitative analysis of smFISH revealed highly focal and variable distributions of interneuron subtypes across the marmoset neocortex (Fig. 6, B to E, and fig. S5 and S6). In both absolute numbers and relative proportions, *PVALB^+^* interneurons were strongly enriched in the occipital lobe, particularly along the calcarine sulcus in the medial sections, as well as the occipital pole more laterally (Fig. 6C, fig. S5 and S6, and data S2). *SST^+^* interneurons in the neocortex increase mediolaterally (Fig. 6, B and C; fig. S5; data S2), but closer inspection revealed that this is driven not by a spatial gradient so much as by highly focal enrichments around primary motor area (M1) and primary somatosensory cortex (S3, S1/2), the cingulate cortex, entorhinal cortex, and medial PFC (Fig. 6C). *CXCL14^+^* neurons are enriched along the calcarine sulcus medially, as well as in ventral aspects of the occipital cortex more laterally. There are higher proportions dorsomedially in the parietal cortex (Fig. 6C). *VIP^+^* neurons were enriched in PFC and increased laterally at or near somatosensory cortex and posterior parietal cortex (Fig. 6C). *LAMP5^+^* interneurons showed a bias to the top of the cortical layers, consistent with dominant composition of this class as neurogliaform layer 1 types (*10*, *77*, *78*), although this class also contains the *LAMP5/LHX6* type that is found in deeper layers (Fig. 6C) (*10*, *20*).

To better appreciate how these distributions relate to neocortical areas, we used a histologically based marmoset neocortical parcellation (*76*) (Fig. 6, D to F) to bin smFISH interneuron proportions by cortical area (Fig. 6, D and E, and fig. S6 and S7). Small adjacent areas were merged, resulting in 26 areal groupings (Fig. 6F). Overall interneuron proportions relative to all cells varied by 4.5-fold across areal groupings (Fig. 6, D and E, and fig. S6B). As a fraction of all cells (DAPI*^+^*), A31 (dorsal posterior cingulate) had the lowest overall proportion of interneurons (3%), while insular cortex (In) had the highest (17%) (Fig. 6D).

In mice, quantitative mapping of interneuron densities has revealed higher *Sst^+^* densities and lower *Pvalb^+^* densities in neocortical areas involved in higher cognitive functions, such as medial frontal and lateral association areas (*22*). This local circuit feature follows cortico-cortical connectivity network topography in mouse (*79*). Conversely, lower *Sst/Pvalb* ratios are associated with mouse primary motor and sensory areas, which are associated with less distributed (and more local) cortical connectivity (*22*). To determine whether primate neocortex followed similar or distinct rules of interneuron allocation, we examined normalized proportions of the four largely mutually exclusive interneuron classes (*PVALB^+^, SST^+^, LAMP5^+^, VIP^+^*) within our marmoset areal groupings (Fig. 6, D and E, and fig. S7A). Lateral temporal cortex, including A36, had the highest *SST/PVALB* ratio. Other areas with high *SST/PVALB* ratios included piriform cortex (Pir), M1, and several medial/orbitofrontal areas (A25, A13, A32). This suggests that some marmoset association areas, notably medial frontal area and lateral temporal cortex, have high *SST/PVALB* ratio composition consistent with higher-order association networks in mice (fig. S7A) (*22*).

Strikingly, other marmoset higher-order areas, including polar and lateral prefrontal areas (particularly A8, A46, A10, A47, and A45), which are thought to be among the most evolutionarily divergent relative to other prefrontal areas (*80*), and which in primates are characterized by long-range cortico-cortical connectivity to other association cortices (*12*, *81*, *82*), do not exhibit high *SST/PVALB* ratios (Fig. 6, D and E, and fig. S7A). Of all cortical areal groupings we measured, the lowest *SST/PVALB* ratio was found in A45, a higher-order lateral prefrontal area with extensive projections to all lobes of the neocortex (*82*). These results suggest that marmoset lateral prefrontal areas may be characterized not by exceptional *SST/PVALB* ratios (Fig. 6, D and E, and fig. S7A). Instead, lateral prefrontal areas have the highest total fraction of *VIP^+^* and *LAMP5/GAD1^+^* interneurons (all above 50% of all interneurons); both of these populations predominately arise from the CGE, a progenitor zone that has expanded in primate evolution (*83*). These results suggest that primate lateral prefrontal areas do not follow the compositional principles that typify frontal areas in the mouse (*22*) or medial frontal areas in marmoset (Fig. 6, D and E, and fig. S7A).

### Interneuron proportions in marmoset striatum follow mediolateral gradients

The striatum, a crucial brain structure involved in motor control, reward, and decision-making, displays complex functional topography and connectivity. In humans, anterio-ventromedial portions containing nucleus accumbens/ventral striatum are functionally coupled to limbic and higher-order cognitive networks, while lateral subdivisions are more coupled to sensory and motor networks (*84*). Prior work relating bulk gene expression measurements in human and macaque striatum to cortico-striatal network organization found a relationship between functional domain (e.g., somato/motor versus limbic network) and regional differences in gene expression, including enrichment of *PVALB* in lateral portions of the striatum associated with sensory and motor processing (*85*). These results suggest that differences in local cell type composition across striatum may underlie aspects of its functional topography.

In mice, striatal interneuron subtypes have distinct spatial distributions: Cholinergic (*Chat^+^*) neuron proportions increase dorsally and anteriorly (*86*), *Pvalb^+^* interneurons are more abundant in dorsolateral striatum than in dorsomedial striatum, and *Sst^+^* interneurons are spatially homogeneous (fig. S7C) (*87*). Primates retain the major populations of striatal interneurons found in mice (*4*, *88*), and additionally have gained the type distinguished by *TAC3* expression (*10*, *46*) described in previous sections (Fig. 7A). A systematic quantification of striatal interneuron types has not been performed comprehensively in a primate, and it is unknown if they follow similar or distinct spatial distributions observed in mice. We therefore used smFISH to investigate distributions of the major types of conserved striatal interneurons (*SST*, *PVALB*, *SLC5A7*/*CHAT*, *TH*, *CCK*, *TAC3*; probes in table S4) in serial sagittal sections of marmoset striatum (Fig. 7B).

Each striatal interneuron population exhibited a nonuniform distribution across the marmoset striatum, particularly in the medial-lateral axis. Similar to mice, the proportion of striatal *PVALB^+^* interneurons increases in lateral sections, from ~0% to 0.8% of all cells (Fig. 7C and data S2). Unlike mice, marmoset *SST^+^* interneuron distribution is nonuniform, appearing sparse near the midline and increasing in proportion (0.1 to 0.7%; Fig. 7C and data S2). Cholinergic neurons (*CHAT^+^*) show the opposite medial-lateral gradient (0.45 to 0.1%; Fig. 7C and data S2). Similar to *CHAT^+^* neurons, *TH^+^* striatal interneurons, which are transcriptionally similar to the *PVALB^+^* type, exhibited a decreasing medial-lateral gradient (Fig. 7C and data S2). *CCK^+^* striatal interneurons, which are a minority population in marmoset (*10*) and mouse (*88*), are enriched close to the midline and become much sparser laterally (Fig. 7C and data S2). The *TAC3^+^* interneurons showed a decreasing medial-lateral gradient, similar to *CHAT^+^* neurons (2 to 0.5%; Fig. 7C and data S2). No striatal population exhibited anterior-posterior or dorsal-ventral gradients with the exception of *PVALB* interneurons, which showed a modest dorsal-ventral gradient. Unlike striatal interneurons, SPN proportions were largely uniform across the major axes: *DRD1* and *DRD2*, which distinguish direct and indirect SPNs, respectively, were slightly enriched medially but otherwise exhibited largely uniform distributions across the striatum (Fig. 7C). Overall, these findings suggest that the spatial distribution of interneuron subtypes within the primate striatum may play a role in shaping its functional topography and connectivity. While mice and primates share similar striatal interneuron populations, there are systematic differences in their spatial distributions.

### Reconstructions reveal regional variation in interneuron morphology

The morphology of interneuron types relates essentially to their function and contribution to neural circuits. While methods such as biocytin filling and Golgi staining are the gold standard for morphological reconstructions, low-titer AAVs carrying membrane-bound fluorescent reporters can be used to sparsely label cells for morphological reconstruction. We used a reporter AAV under the control of the forebrain interneuron-specific mDlx enhancer (*29*) to label neocortical and striatal interneurons, and then performed smFISH (probes in table S4) on thick sections (120 μm) to confirm the molecular identity of GFP*^+^* cells. Reconstructions using viral labeling are more challenging than with single-cell filling methods, because GFP expression from neighboring cells and passing fibers has to be distinguished from signal attributable to the target cell. In some cases, the GFP signal appears punctate, making it challenging to follow discontinuous processes. For these reasons, our reconstructions are conservative: We aimed to avoid reconstructing false-positive fibers (fibers originating from other cells) and, in some cases, likely under-ascertain the full dendritic arborization of target cells.

**Fig. 8.**
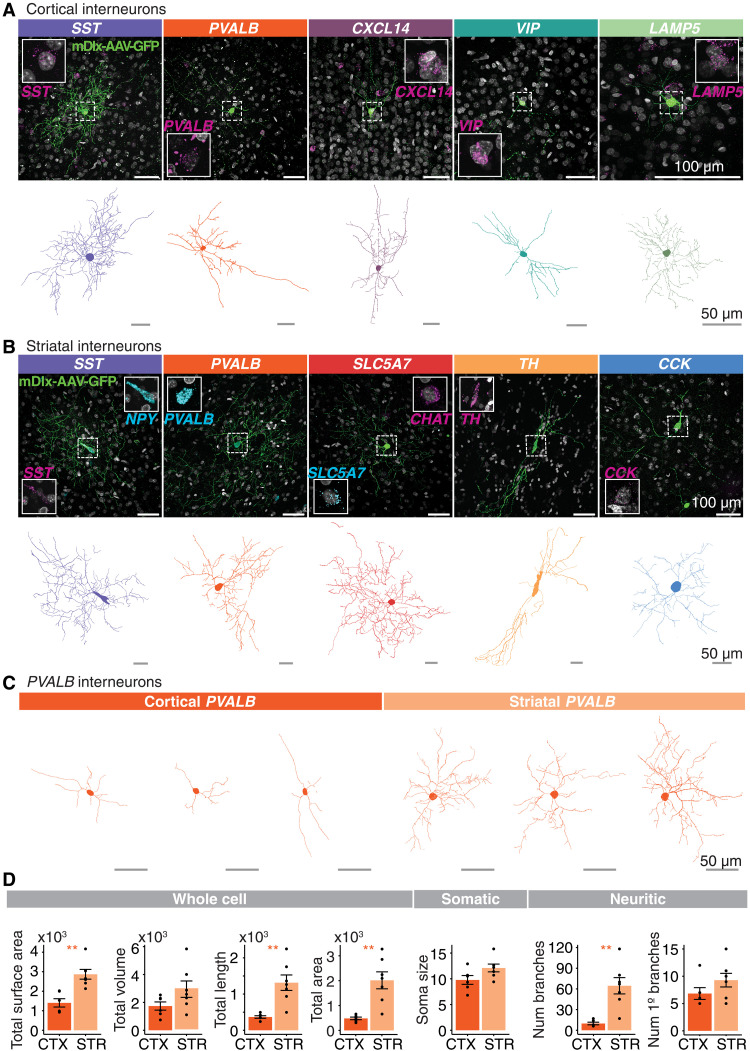
Morphological characteristics of marmoset cortical and striatal interneurons. (**A** and **B**) Top rows: Examples of AAV9-hDlx5/6-GFP-fGFP labeled neocortical (A) and striatal (B) interneurons. Insets show magnified cell nucleus of GFP^+^ cell along with smFISH staining for interneuron type marker to confirm molecular identity of labeled cell. Scale bar, 100 μm. Bottom rows: Reconstructed skeletonized morphology (Imaris) of GFP^+^ cells depicted in the top rows. Scale bar, 50 μm. (**C**) Representative reconstructed neocortical and striatal *PVALB*^+^ interneurons from one marmoset (Cj 17-154; tables S1 and S6). Scale bar, 50 μm. (**D**) Quantification of morphological characteristics of *PVALB*^+^ cells from Cj 17-154. Means ± SEM. *P < 0.05 and **P < 0.01. See Materials and Methods for detailed statistical information.

We imaged 1203 GFP*^+^* neurons in the striatum and 4374 GFP*^+^* neurons in the neocortex. We used NeuTube to reconstruct the top 216 telencephalic neurons that had the qualitatively highest signal to noise and most complete soma and processes based on GFP signal, did not have other cells labeled in the field of view (FOV), and were positive for at least one smFISH probe (table S5; examples in fig. S8). Raw image stacks and NeuTube reconstructions are available from the BIL. Using combinations of one to two probes for marker genes of different types, we identified GFP*^+^* neocortical and striatal interneurons, respectively, with smFISH (neocortex: *SST*, *PVALB*, *CXCL14*, *VIP*, *LAMP5*; striatum: *SST*, *PVALB*, *SLC5A7*, *TH*, *CCK*; table S4) (Fig. 8, A and B, top rows). We used a second tracing and reconstruction method (Imaris) on the 41 most complete GFP*^+^* cells. Of these, we discarded 5 due to incomplete soma or highly discontinuous processes in the image stack, retaining 36 cells (Fig. 8, A and B, bottom rows, and table S6). Morphological parameters were measured using the Surface function, which detects surface area and volume based on the fluorescence of the mDlx-AAV-GFP expression, and the Filament Tracer function, which traces structural features starting from the soma to the terminal processes based on the diameter of the soma and the thinnest projection of the cell (table S6). To assess whether there are region-dependent morphological differences within molecularly similar types, we compared several parameters between a collection of neocortical and striatal *PVALB^+^* GFP*^+^* cells that were reconstructed from the same marmoset (Cj 17-154; Fig. 8, C and D) to avoid variable tissue shrinkage arising from different tissue storage conditions across the animals (see Materials and Methods). Striatal *PVALB*^+^ GFP^+^ cells were larger than cortical *PVALB*^+^ GFP^+^ cells in terms of length and area, but not soma diameter or the volume of GFP^+^ fluorescence (Fig. 8D). While the number of branches issued by their somas also did not differ, the striatal *PVALB*^+^ GFP^+^ interneurons exhibited more dendritic branch points than their cortical counterparts (Fig. 8D), suggesting that marmoset striatal *PVALB*^+^ interneurons have greater arborization than *PVALB*^+^ cortical interneurons.

### An enhancer-AAV labels a primate-specific interneuron type

Given that the primate-specific *TAC3*^+^ type comprises almost 30% of striatal interneurons in marmoset (*10*), we expected a sizable proportion of striatal GFP^+^ cells labeled by AAV9-hDlx5/6-GFP-fGFP to be *TAC3*^+^. However, while 55 GFP^+^ striatal cells were imaged across all smFISH *TAC3* probe–treated sections, we failed to find any colocalization of GFP with *TAC3* transcripts. In these experiments, which were double-labeled with *TAC3* and *PVALB* probes, 9 of 55 were GFP^+^, *PVALB*^+^ (16% *PVALB*^+^, versus expected 21% of interneurons expected from snRNA-seq proportions), while 0 of 55 were GFP^+^, *TAC3*^+^ (0% versus 28% expected from snRNA-seq proportions).

**Fig. 9.**
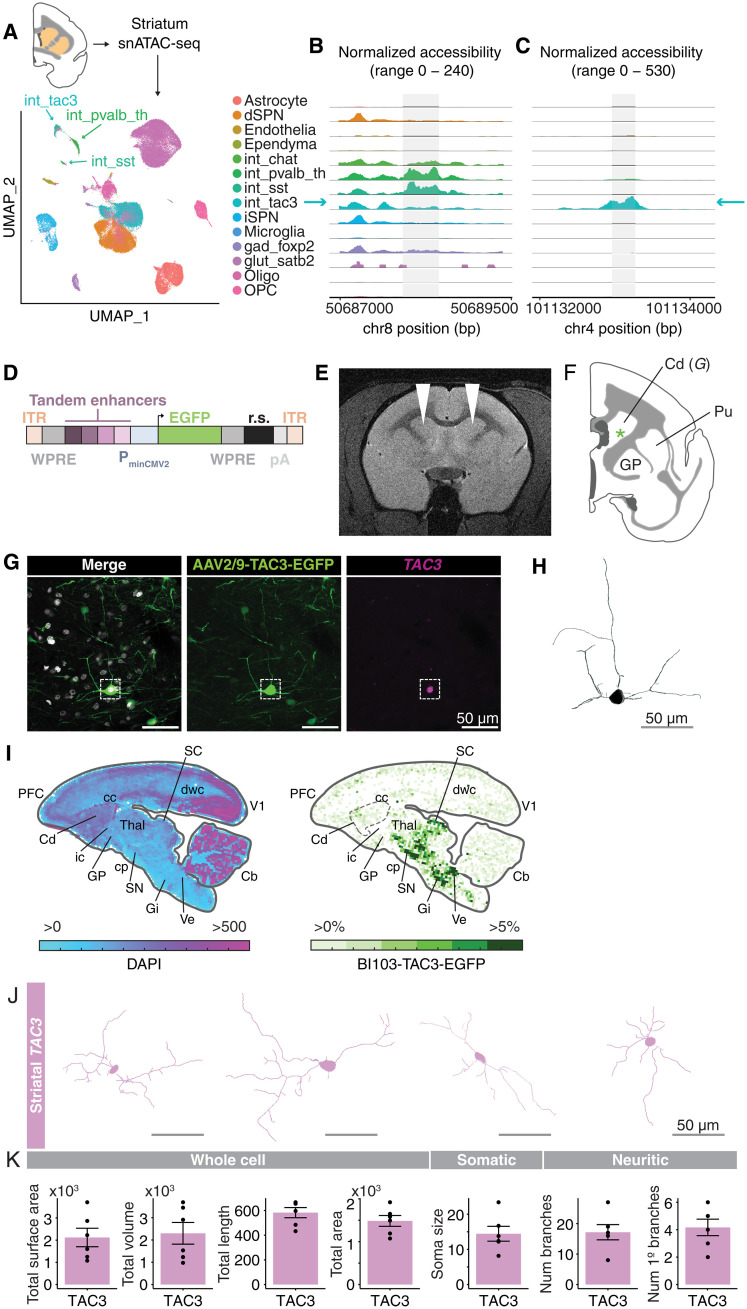
Development of an enhancer-AAV for *TAC3*primate-specific striatal interneurons. ^+^ (**A**) snATAC-seq (69,808 nuclei) from fresh marmoset striatum (Cj 18-153; table S1). Uniform Manifold Approximation and Projection (UMAP) with labels transferred from striatal snRNA-seq. (**B**) *TAC3*^+^ interneurons lack accessibility at the locus corresponding to the mDlx sequence (*89*) in marmoset. (**C**) Chromatin accessibility of a candidate enhancer for the *TAC3*^+^ type. (**D**) Construct design for *TAC3* interneuron AAV. Four candidate regulatory elements [example in (C)] were packaged in tandem upstream of a minimal promoter driving EGFP expression. ITR, inverted terminal repeats; WPRE, Woodchuck hepatitis virus posttranscriptional regulatory element; P_minCMV2_, minimal CMV2 promoter; EGFP, enhanced green fluorescent protein; r.s., 300 bp random sequence; pA, polyadenylation sequence. (**E**) MRI showing injection location (white arrowheads) of the AAV9-tandemE-TAC3-EGFP virus into dorsal striatum in one animal (Cj 19-207; table S1). (**F**) Location of cell shown (G). (**G**) Main: EGFP antibody–amplified image of a labeled cell [position in (F)]. Insets: smFISH of *TAC3* colocalization. Scale bar, 50 μm. (**H**) Morphological reconstruction (Imaris) of cell in (G). Scale bar, 50 μm. (**I**) Heatmaps showing the density of cells transduced by AAV-BI103-tandemE-TAC3-EGFP after systemic intravenous injection. Left, density of all DAPI^+^ nuclei per unit area profiled (approximately 344 μm per bin); right, density of EGFP^+^ cells relative to DAPI^+^ cells. PFC, prefrontal cortex; Cd, caudate; cc, corpus callosum; ic, internal capsule; GP, globus pallidus; Thal, thalamus; cp, cerebral peduncle; SN, substantia nigra; Gi, gigantocellular reticular nucleus; SC, superior colliculus; dwc, deep cortical white matter; Ve, vestibular nucleus; V1, visual cortex V1; Cb, cerebellum. (**J**) Reconstructions (Imaris) of striatal EGFP^+^/*TAC3*^+^ cells. (**K**) Morphological parameters of reconstructed cells from striatal EGFP^+^/*TAC3*^+^ cells from local injection (E) and systemic injection (I) (table S6).

The mDlx enhancer is a regulatory element specific to forebrain interneurons (*89*). Although the regulatory element, and the forebrain-interneuron expressed genes flanking it, *Dlx5* and *Dlx6*, are highly conserved in vertebrates, we wondered whether the lack of accessibility of the mDlx locus in the *TAC3* interneurons could explain our inability to find colocalization of *TAC3* expression and GFP. To assess this, we generated snATAC-seq data (69,808 nuclei) from fresh marmoset striatum (Fig. 9A). We used Signac (*90*) to integrate our previously annotated striatal snRNA-seq data and identify major striatal types. We then examined accessibility of the marmoset sequence homologous to the mDlx locus across interneuron types. While other striatal interneuron types (particularly the *SST*^+^ and *PVALB*^+^ types) showed accessibility (ATAC-seq peaks, reflecting chromatin accessibility) at the mDlx locus, the *TAC3*^+^ type did not (Fig. 9B).

To develop a viral tool that could transduce the striatal *TAC3*^+^ cell type, we next nominated candidate regulatory elements specific to the *TAC3*^+^ interneuron type. We used Signac to identify differentially accessible peaks in the *TAC3*^+^ cluster relative to all others and filtered the set by fold change, percent accessibility across the target cell type population, and peak size (Fig. 9C). To maximize the likelihood of obtaining a functional reporter while minimizing the number of marmosets used, and because several of our top candidates were very short, we selected four top regulatory element candidates for the *TAC3*^+^ type for tandem packaging (see Materials and Methods). The four candidates were located on four different chromosomes and spanned between 94 and 215 base pairs (bp). One site was in an exon of *CDH13*, one was in the first exon of *TAC3*, one was in an exon of *LOC108592287*, and one was intergenic (closest gene 200 kb distance). These four elements were packaged in tandem in an AAV9 vector (AAV9-tandemE-TAC3-EGFP) containing a cytoplasmic enhanced GFP (EGFP) reporter (Fig. 9D). We delivered the virus via magnetic resonance imaging (MRI)–guided local injection into the anterior striatum of two marmosets (Cj 19-207 and 17-B111; Fig. 9E, fig. S9A, and table S1) and imaged coronal sections of striatum for EGFP-positive cells after 6 and 10 weeks of incubation time (Fig. 9F and fig. S9B). smFISH confirmed colocalization of *TAC3* transcripts in EGFP^+^ neurons in striatum (Fig. 9G and fig. S9, C and D). In one animal, the virus diffused beyond the boundary of the striatum. In this animal, we also detected strongly labeled EGFP^+^ cells in a border zone between striatum, BNST (bed nucleus of the stria terminalis), and the globus pallidus. smFISH confirmed that these cells, too, were *TAC3*^+^ (fig. S9, B to E).

To determine the broader biodistribution of cells transduced by the tandemE-TAC3 enhancer, we next packaged the same enhancers in an AAV capsid (BI103) capable of efficient transduction of brain cell types after systemic intravenous delivery in marmosets. We delivered this AAV (AAV-BI103-tandemE-TAC3-EGFP) to one marmoset (Cj 20-214; table S1). After a 4-week incubation, the brain was perfused, sliced, and stained with anti-GFP antibody to amplify EGFP signal, and with smFISH probes against *TAC3*^+^ to confirm colocalization. We found EGFP^+^/*TAC3*^+^ colocalized cells in striatum as well as several extra-striatal locations including hypothalamus, substantia nigra, superior colliculus, brainstem, and neocortex (Fig. 9I and fig. S9, F and G). To assess the morphology of *TAC3*^+^ striatal interneurons produced by viral labeling, we reconstructed several of the most complete cells from both the local injections (AAV9-tandemE-TAC3-EGFP) and the systemic injection (AAV-BI103-tandemE-TAC3-EGFP) using Imaris (Fig. 9, H, J, and K, and table S6). The cells tended to have two to three thick branches that extended from the soma, and which bifurcated close to the soma and became thinner thereafter. Reconstructed cells had median 13.1 μm (± 5.1 SD) soma diameter, 2221.3 volume (μm^3^; ± 1192.8 SD), and 16.5 (± 6.11) total dendritic branch points. These experiments show that whereas systemic injections of AAV9 under the mDlx enhancer could not transduce the striatal *TAC3*^+^ type, presumably due to lack of accessibility at the mDlx locus in this cell type (Fig. 9B), local injections of AAV9 as well as systemic injections with alternative capsid with the cell type–targeted enhancers were successful. Broadly, the use of cell type–specific regulatory elements, coupled with viral engineering, enables a new frontier for the study of highly divergent primate brain cell types.

## DISCUSSION

Here, we generated a molecular and cellular census of the adult marmoset brain using snRNA-seq, quantitative smFISH, and cell type–specific AAV labeling. Our study reveals the complex repertoire of cell types in the marmoset brain. Our snRNA-seq dataset of over 2.4 million brain cells across 18 brain regions in the marmoset indicates that lineage is an important factor shaping adult transcriptomic identity of neuronal types, apparently more so than neurotransmitter utilization (Figs. 1 to 5). Using quantitative smFISH, we revealed, for the first time in a primate, the spatial distributions of molecularly resolved GABAergic interneuron types (Figs. 6 and 7). Using GFP delivered by interneuron-specific AAVs, we generated morphological reconstructions of all major interneuron types in the neocortex and striatum (Figs. 8 and 9). We generated a viral genetic tool under the control of cell type–specific regulatory elements to successfully label a previously described putative primate-specific striatal interneuron type (Fig. 9). These datasets, generated as part of the BICCN, complement other recent and extensive cellular profiling studies in other species (*1*, *2*, *30*–*32*) and will enable insights in cell type innovations and modifications in future comparative studies.

Telencephalic glutamatergic and GABAergic neurons strongly segregate in mammals as well as in homologous structures in amphibians (*8*), suggesting an evolutionarily conserved distinction. An initial atlas in mouse indicated that glutamatergic and GABAergic neurons from diencephalic and midbrain structures also partition almost perfectly by neurotransmitter usage (*5*), suggesting that this could be a general rule. However, more recent transcriptomic censuses in mouse (*1*, *30*) and human (*2*) indicate that across mammalian species, many nontelencephalic glutamatergic and GABAergic types tend to form highly intermixed clades that do not separate clearly by neurotransmitter identity. Our results in the adult marmoset (and mouse; Fig. 3) concord with the notion that gene expression distinctions between telencephalic glutamatergic and GABAergic neurons do not hold for neurons in other brain structures (Figs. 1 to 3). This has implications for generalizing transcriptomic associations to other phenotypes. For example, transcriptomic changes in glutamatergic or GABAergic neurons have been associated to diseases such as autism and schizophrenia (*91*–*93*). Such associations may not generalize to GABAergic or glutamatergic types outside of the sampled brain region (usually neocortex), consistent with observations that “global” changes to glutamatergic or GABAergic neurons in relation to disease actually often only surface in a few brain regions (*94*).

A previous analysis, based on shared patterns of several key TFs, proposed that telencephalic GABAergic neurons are developmentally and evolutionarily related to diencephalic GABAergic neurons (*95*). Our results indicate that when profiled at adulthood, only a limited number of telencephalic GABAergic types are transcriptionally similar to diencephalic types, some of which may actually arise from cephalic boundary crossings earlier in development. Most neocortical, hippocampal, and some amygdalar and striatal GABAergic types are so distinct from diencephalic GABAergic types that they share more gene expression in common with telencephalic glutamatergic types (Fig. 1C). In general, the hierarchical arrangement of neuron types (the neuronal taxonomy) across brain structures was preserved across marmoset and mouse datasets (Fig. 3C), suggesting an evolutionarily conserved arrangement. An important future task is to leverage recent brain cell census datasets in macaque (*31*) and human (*2*) to investigate the consistency of these observations over a broader range of species.

Developmental origin or shared lineage plays a strong role in shaping the adult transcriptomic identity of neurons, but phenotypic convergence, whereby adult cell types converge on similar transcriptomic identities despite a nonshared developmental origin, may also drive apparent similarities among cell types. Recent advances in lineage tracing coupled with single-cell RNA sequencing demonstrates that phenotypic convergence is surprisingly common between transcriptomically defined types (*19*, *74*). As such, it is difficult to disambiguate between these possibilities in the absence of data that confirm lineage directly (*8*, *19*). For example, in our data, the clade of GABAergic projection neurons that contains striatal SPNs and amygdala and basal forebrain *GAD1*^+^*, FOXP2*^+^ neurons also contained several subtypes of hypothalamic *GAD1*^+^*, FOXP2*^+^ neurons (Figs. 1C and 4 and fig. S2D). Either these hypothalamic *FOXP2*^+^ subtypes have a convergent expression identity with long-range GABAergic projection neurons of the telencephalon or they arise from a common lineage.

Our analysis of nonneuronal cells revealed striking regional heterogeneity of astrocytes across major brain divisions, to a greater extent than oligodendrocytes, OPCs, microglia, ependymal cells, and vascular cells (Fig. 1D and fig. S2). Regional heterogeneity of astrocytes is well established in the mouse (*5*, *96*–*98*) and has recently been demonstrated in marmoset (*18*) and human (*2*) transcriptomic studies. The division of astrocyte populations across cephalic boundaries, and their differential expression of early patterning genes such as *FOXG1* [fig. S2, F to H; also (*18*)], suggests a developmental origin for astrocyte regional heterogeneity [discussed in (*67*)], perhaps as early as the first trimester in humans (*99*). Our atlas builds upon these findings by providing increased resolution of intra-cephalic regional heterogeneity in astrocytes, which we explored in the cortex. We found moderate proportional differences in cortical area representation between astrocyte and oligodendrocyte clusters (Fig. 5, C to E), except for an astrocyte subtype (cluster 3) marked by high *VCAN* expression that localized to V1-adjacent white matter. Notably, astrocytes and oligodendrocytes within the cortex exhibited unique regional gene expression signatures between cortical areas that were not shared with neurons, despite a common developmental origin between cortical glutamatergic neurons and macroglia (*71*, *99*–*102*).

Our data support known instances of cross-cephalic migrations in thalamus and amygdala, and also suggest new ones (Fig. 4). The similarity of the primate-specific *TAC3*^+^ striatal type to hypothalamic and basal forebrain *TAC3*^+^ types is unexpected (Fig. 4H) and suggests another possible cross-cephalic vesicle migration event. Recent work (*46*) proposed that the striatal *TAC3*^+^ type has a ventral ventricular telencephalic origin, similar to most other GABAergic interneuron types destined for striatum. While phenotypic convergence remains possible, another possibility is that a ventral telencephalic progenitor gives rise to both telencephalic and diencephalic types. This is supported by the expression of *FOXG1*, a TF necessary for ventral telencephalic fate (*103*), in all three transcriptionally similar *TAC3*^+^ populations. While cross-cephalic migration events may be nominated on the basis of unexpected transcriptional similarity, as we did here using hierarchical clustering analyses (Figs. 3 and 5 and fig. S4), ultimately these and other instances of unexpected transcriptional similarity should be investigated using lineage tracing or other methods capable of inferring developmental relationships directly.

Developmentally linked interneuron populations in striatum and neocortex (e.g., *PVALB*^+^ types in both structures) displayed distinct spatial distributions measured by cell counting of smFISH imaged across serial sections of the marmoset brain (Figs. 6 and 7). While all interneuron subtype distributions in the striatum followed an increasing or decreasing gradient along a medial-lateral axis, the interneurons in the neocortex followed more complex distributions that were not captured by simple gradients. In the mouse, *Sst*^+^*/Pvalb*^+^ ratios are a hallmark of higher-order cortex (*22*), but we found a different pattern across much of the higher-order association cortex in marmosets. Areas in the marmoset lateral PFC were unique in their relatively high proportions of *VIP*^+^ neurons (and had unexceptional or even low ratios of *SST*^+^/*PVALB*^+^ neurons) (Fig. 6 and figs. S5 and S6). These results suggest that primate lateral prefrontal areas may not follow the same local cortical circuit organizing principles that pertain to mouse association cortex.

Morphological characterization of the striatal and neocortical interneuron populations suggests variation in overall size and dendritic arborization among subtypes (Fig. 8). Striatal *PVALB*^+^ cells were found to be larger in length, volume, and surface area and consisted of more dendritic branch points than the cortical cells. Our data altogether suggest that the striatal *PVALB*^+^ interneurons exhibit higher dendritic complexity compared to their respective counterparts in the neocortex (Fig. 8, C and D). These morphological differences could result in differing electrophysiological properties. For example, it is possible that *PVALB*^+^ cells in the striatum have higher capacitance and receive more synaptic inputs onto them, thereby impacting local signal integration and computation, than cortical *PVALB*^+^ interneurons. The functional importance of morphological differences across the interneuron subtypes identified in our study will ultimately require studying how these cells affect the circuits in which they reside (i.e., cellular/subcellular targeting biases and functional properties) (*27*, *104*, *105*).

Recent bioengineering advances have improved delivery of viruses across the blood-brain barrier to enable cell type–specific access in nonhuman primates (*17*, *29*, *106*–*109*). To maximize translatability across species, many approaches that use cell type–specific regulatory elements to restrict expression rely on evolutionary conservation (e.g., between mice and humans) as a screening criterion. However, the evolutionarily conserved mDlx enhancer, which generally labels telencephalic interneurons in a range of mammalian species, did not drive expression in the primate-specific striatal *TAC3*^+^ cell type (Fig. 9B). This could be due distinct developmental origins (Fig. 4, H and I), or because the *TAC3*^+^ type evolved along the primate lineage and accumulated a divergent regulatory landscape. However, we also observed that the mDlx enhancer selectively under-ascertained several other interneuron populations, and often in region and cell type–specific ways. For example, it systematically under-labeled *VIP*^+^ and *SST*^+^ types in the neocortex (expected 22% and 26% of interneurons, obtained 2% and 3%, respectively), as well as *SST*^+^ interneurons in the striatum (14%, obtained 1.8%). This under-labeling could in part be due to the titer and systemic delivery approach we adopted, which was necessary to achieve sparse labeling for morphology.

The lack of transduction of *TAC3*^+^ striatal interneurons using the AAV-mDlx virus prompted us to develop an AAV (AAV9-tandemE-TAC3-EGFP) under the control of candidate *TAC3*^+^ interneuron striatal regulatory elements (Fig. 9, D to H). This enabled initial reconstructions of striatal TAC3^+^ interneuron morphology (Fig. 9, H and J). When delivered systemically using a capsid that has efficient transduction across the blood-brain barrier in marmosets (Bi103-tandemE-TAC3-EGFP), the virus also labeled *TAC3*^+^ neurons elsewhere in the brain, including in the hypothalamus, neocortex, and substantia nigra. Neocortical *TAC3*^+^ cells bear little resemblance to *TAC3*^+^ striatal interneurons in terms of their global gene expression profiles (Fig. 4H). Therefore, the widespread transduction of *TAC3*^+^ neurons suggests that one or more of the regulatory elements that we used (originally defined only relative to other striatal cell types) may endogenously regulate the *TAC3* gene itself, or else may control another gene that tends to be commonly expressed in developmentally and transcriptionally disparate *TAC3*^+^ populations. Further refinements to these AAVs can optimize for specificity or completeness for the striatal TAC3^+^ type alone according to experimental goals and delivery strategy.

Together, our study reveals the complex landscape, biodistribution, and morphological variation of transcriptionally defined cell types in the marmoset brain. The persistent fingerprint of developmental origin present in neuronal gene expression underscores important roles for novel neurogenic niches, developmental patterning, and altered mechanisms guiding proliferation and cell migration in primate brain evolution that could be the subject of future study. These resources will enable further comparative studies of the evolution and development of primate brain cell types.

## MATERIALS AND METHODS

### Animals used for study

#### Marmosets

Marmosets were pair-housed in spacious holding rooms with environmental control of temperature (23° to 28°C), humidity (40 to 72%), and 12-hour light/dark cycle. Their cages were equipped with a variety of perches and enrichment devices, and they received regular health checks and behavioral assessment from Massachusetts Institute of Technology (MIT) Division of Comparative Medicine (DCM) veterinary staff and researchers. All animal procedures were conducted with prior approval by the MIT Committee for Animal Care (CAC) and following veterinary guidelines.

#### Mice

Experimental mice were purchased from The Jackson Laboratory company and housed at the McLean hospital animal facility (three to five mice per cage) on a 12:12-hour light-dark cycle in a temperature-controlled colony room with unrestricted access to food and water. All procedures were conducted in accordance with policy guidelines set by the National Institutes of Health and were approved by the McLean Institutional Animal Care and Use Committee (IACUC).

### Tissue processing for single-nucleus sequencing and smFISH

#### Marmoset specimens for snRNA-seq

Marmoset experiments were approved by and in accordance with MIT CAC protocol number 051705020. Adult marmosets (2 to 14.5 years old, 12 individuals; table S1) were deeply sedated by intramuscular injection of ketamine (20 to 40 mg kg^−1^) or alfaxalone (5 to 10 mg kg^−1^), followed by intravenous injection of sodium pentobarbital (10 to 30 mg kg^−1^). When the pedal withdrawal reflex was eliminated and/or the respiratory rate was diminished, animals were transcardially perfused with ice-cold sucrose-Hepes buffer (*4*, *10*). Whole brains were rapidly extracted into fresh buffer on ice. Sixteen 2-mm coronal blocking cuts were rapidly made using a custom-designed marmoset brain matrix. Slabs were transferred to a dish with ice-cold dissection buffer (*4*, *10*). All regions were dissected using a marmoset atlas as reference (*110*), snap-frozen in liquid nitrogen or dry ice–cooled isopentane, and stored in individual microcentrifuge tubes at −80°C.

#### Marmoset specimens for snATAC-seq

Tissue from one marmoset (female, 2 years old; table S1) was used for both snRNA-seq and snATAC-seq (table S1). Fresh tissue was dissected from anterior striatum (including caudate and putamen) and used immediately for snATAC-seq.

#### Mouse specimens for amygdala snRNA-seq

Three adult (P80 to P90) male wild-type mice were deeply anesthetized with isoflurane and sacrificed by decapitation. Brains were quickly excised, washed in ice-cold sterile 0.1 M phosphate-buffered saline (PBS), and dissected onto an ice-cold glass surface. Amygdala nuclei were identified and isolated using “The Allen Mouse Brain Atlas” (https://mouse.brain-map.org/static/atlas; table S7) as a reference for anatomical landmarks. The basolateral amygdaloid nucleus was exposed by performing two coronal cuts using the borders of primary somatosensory cortex and primary visual cortex as landmarks. Dissected specimens were collected in 1.5-ml microcentrifuge tubes, snap-frozen on dry ice, and stored at −80°C until use.

#### Marmoset specimens for smFISH

Two marmosets were deeply sedated by intramuscular injection of alfaxalone (5 to 10 mg kg^−1^) (table S1), followed by intravenous overdose of sodium pentobarbital (10 to 30 mg kg^−1^). When the pedal withdrawal reflex was eliminated and/or the respiratory rate was diminished, animals were transcardially perfused with ice-cold saline. The brain was immediately removed, embedded in optimal cutting temperature (OCT) freezing medium, and flash-frozen in an isopropyl ethanol–dry ice bath. Samples were cut on a cryostat (Leica CM 1850) at a thickness of 16 μm, adhered to SuperFrost Plus microscope slides (VWR, 48311-703), and stored at −80°C until use. Portions of the brain that were not cut were recoated in OCT and stored again for future use. Samples were immediately fixed in 4% paraformaldehyde (PFA) and stained on the slide according to the Molecular Instruments HCR generic sample in solution RNA-FISH protocol (Molecular Instruments, https://files.molecularinstruments.com/MI-Protocol-RNAFISH-GenericSolution-Rev7.pdf; table S7) or the Advanced Cell Diagnostics RNAscope Multiplex Fluorescent Reagent Kit v2 Assay (ACD; 323100, https://acdbio.com/sites/default/files/UM%20323100%20Multiplex%20Fluorescent%20v2_RevB.pdf) protocol (table S7).

### snRNA-seq, library preparation, sequencing

#### 10x RNA-seq

Unsorted single-nucleus suspensions from frozen marmoset and mouse samples were generated as in (*10*). Gel-Bead-in-emulsion (GEM) generation and library preparation followed the manufacturer’s protocol (10X Chromium single-cell 3′ v.3, protocol version #CG000183_ ChromiumSingleCell3′_v3_UG_Rev-A). Raw sequencing reads were aligned to the National Center for Biotechnology Information (NCBI) CJ1700 reference (marmoset) or GRCm38 (mouse). Reads that mapped to exons or introns were assigned to annotated genes. Libraries were sequenced to a median read depth of 8 reads per unique molecular identifier (UMI; or transcript), obtaining a median 7262 UMIs per cell.

### RNA sequencing data processing, curation, clustering

Processing and alignment steps follow those outlined in the Drop-seq alignment workflow (table S7). Raw BCL files were processed using IlluminaBasecallsToSam, and reads with a barcode quality score below Q10 were discarded. Cell barcodes (CBCs) were filtered using the 10X CBC whitelist, followed by template switching oligo (TSO) and poly(A) trimming. Reads were aligned using STAR and tagged with their gene mapping [exonic, intronic, or untranslated region (UTR)] and function (strand). The reads were then processed through GATK BQSR and tabulated in a digital gene expression (DGE) matrix containing all CBCs with at least 20 transcripts aligning to coding, UTR, or intronic regions. Cell selection was performed based on CellBender remove-background non-empties (*111*), % intronic (% of a CBC’s reads that are intronic), and number of UMIs for a CBC. A new filtered DGE containing only these selected CBCs was then generated. Finally, a gene-metagene DGE was created by merging the selected CBCs DGE with a metagene DGE (made by identifying reads from the selected CBCs that have a secondary alignment mapping to a different gene than its primary alignment).

Cell type classification models were trained using our annotations and scPred R package version 1.9.2 (*35*). Detection of cell-cell doublets was performed using a two-step process based on the R package DoubletFinder (*112*). DoubletFinder implements a nearest-neighbors approach to doublet detection. First, artificial doublets are simulated from input single-cell data and are coclustered with true libraries. True doublet libraries are identified by their relative fraction of artificial doublet nearest-neighbors in gene expression space. In our workflow, this process is run twice, once with high stringency to identify and remove clear doublets, and again with a lower threshold to identify remaining subtler doublet libraries. We used the following parameters for round 1: PN = 0.4, PK = 10 ^ seq(-4, -1, length.out = 50), NUM_PCS = 5. For the second round: PN = 0.45, NUM_PCS = 10, PK = 10 ^ seq(-4, -1, length.out = 50). Because doublet libraries are initially categorized as true libraries in the nearest-neighbor search, we find that this two-step process improves the sensitivity and accuracy of doublet detection.

Clustering was performed using independent component analysis (ICA; R package fastICA) dimensionality reduction followed by Louvain. Cells assigned to one of the major glial types (oligodendrocyte lineage, astrocytes, vascular/endothelia, microglia/macrophages) by scPred were collected across all brain regions and clustered together. Neurons from most telencephalic structures (neocortex, hippocampus, striatum, amygdala) confidently assigned to the categories “GABAergic” and “glutamatergic” and so were clustered separately by neurotransmitter usage for each brain structure. Striatal neuron categories were “SPN” and “GABAergic interneuron.” Neurons in nontelencephalic brain structures were clustered separately by brain structure. All clusterings were performed in two stages: First-round clustering was based on the top 60 ICs and a default resolution (res) of 0.1, nearest neighbors (nn) = 25. Following the process outlined in (*4*), each was manually reviewed for skew and kurtosis of gene loadings on factors and cells to identify ICs that loaded on outliers, doublets, or artifactual signals. These were discarded, and reclustering was performed on the remaining ICs. Each resulting cluster was then subjected to second-round clustering, during which ICs were again curated. Second-round clustering explored a range of parameters: nn = 10, 20, 30; res = 0.01, 0.05, 0.1, 0.2, 0.3, 0.4, 0.5, 1.0. Final parameter values were chosen to optimize concordance, when possible, between the final number of clusters and the number of included ICs such that each cluster was defined by one primary IC. Metacells for each cluster are generated by summing transcript counts for each cell across all cells in the cluster, normalizing by total number of transcripts, and scaling to counts per 100,000. Hierarchical clustering of metacells was performed in two ways: (i) on the top 100 PCA components (HCA PCA) (using the HCPC package), and (ii) using Pearson correlation as the distance function (HCA Corr). Trees were visualized using the ggtree package suite (*113*). See associated code for plotting functions (table S7).

### Identification of rDEGs

We computed rDEGs for neocortical glutamatergic neurons, GABAergic neurons, astrocytes, and oligodendrocyte lineage types. We used individual cell level cluster assignments from the initial neocortical clustering (which contained all regions together) to create per-region metacells for each cluster. Normalized metacells were log_10_-transformed, and pairwise differences across regions within the same cluster (cell type) were examined. Genes with >3-fold difference in the same cluster between two regions were considered rDEGs. For this analysis, we omitted from comparisons any region-cluster metacell generated from fewer than 50 cells, but retained comparisons between regions that had >50 cells in that cluster. Genes that were consistently rDEGs in at least three individuals for each cluster-region pair are reported in table S3.

### Ancestral state reconstruction

Ancestral state reconstruction (ASR) is a method to infer hidden ancestral traits from extant observations. For example, given a phylogenetic tree of species and genomic sequences thought to be homologous across those species, the reconstruction takes into account branch lengths to reconstruct the most likely ancestral sequence. We applied a maximum likelihood–based ASR approach (R package: fastAnc) to the scaled, normalized metacells of cell types and the dendrogram of their similarity to produce estimates of expression of each gene at the internal nodes of the tree. These enabled comparisons of leaf nodes to internal nodes as well as internal nodes to each other. For example, compared to the parent node of amygdala, basal forebrain, and hypothalamic SPN-like GABAergic projection neurons, the reconstructed parent node of striatal SPNs had higher expression of known markers of striatal projection neurons such as *DACH1.* We used these reconstructions of internal node gene expression to compare major clades of the tree, and used a chi-square test to determine whether TFs were overrepresented among differentially expressed genes (threshold: threefold difference) between pairs of internal nodes.

### Spatial smFISH experiments

All probes are listed in table S4. All smFISH validation experiments were carried out on distinct biological replicates from those used for snRNA-seq or single-cell ATAC-seq experiments.

#### smFISH tissue processing and quantification

Two marmosets (Cj 18-134 and 19-212) were euthanized and perfused with PBS (table S1). The brain was removed, embedded rapidly in OCT, and stored in the −80°C freezer. Tissue was then cut to 16 μm on a cryostat and stored in the −80°C freezer until needed. In situ hybridization was performed for genes of interest (see table S4) with HCR or ACD antisense probes and incubated with TrueBlack Lipofuscin Autofluorescence Quencher (Biotium, 23007) for 10 s at room temperature to eliminate confounding lipofuscin autofluorescence present in the tissue. Samples were then coverslipped with ProLong Diamond Antifade Mountant (Invitrogen, P36970). Z-stack serial images were taken through the whole depth across striatum, hypothalamus, and basal forebrain regions, and several regions of neocortex, on the TissueGnostic TissueFAXS SL slide-scanning, spinning disk confocal microscope (Hamamatsu Orca Flash 4.0 v3) using a 20×/0.8 NA (numerical aperture) air objective for ACD stains or a 40×/1.2 NA water-immersion objective for HCR stains.

Series images were segmented using StrataQuest, a software package from TissueGnostics, which enables the quantification of signals within segmented images (similar to CellProfiler). Nuclei objects were generated using the DAPI channel, and artifacts were removed based on size and intensity. Exclusion regions of interest (ROIs) were manually drawn to avoid areas of white matter, large artifacts, and autofluorescence before computing intensity and other statistical and morphological measurements (20 parameters) for each channel. Specifically, 50 cells were hand-labeled as positive or negative for the markers of interest to identify the appropriate threshold for feature selection using the parameters that best discriminated this binary. Parameters included mean intensity, maximum intensity, SD of intensity, range of intensity, and equivalent diameter. These filters were then applied to the unlabeled data to identify positive cells.

Segmented cells were further analyzed using in-house code (https://doi.org/10.5281/zenodo.8364941; table S7). Spatial locations of the cells were visualized by plotting the *x*-*y* coordinates associated with each nucleus. These were then binned into 2D histograms across the *x* and *y* axis (corresponding to the rostrocaudal plane and to the dorsoventral plane, respectively). A size of 100 bins was chosen for the first (medial-most) slice of a series across the *x* axis, and the calculated bin size was then used across the *y* axis of the first slice and across other slices in the series. Positive events for a gene in a given bin were either normalized to the number of detected nuclei in that bin (DAPI) and plotted in 2D as a relative heatmap or simply plotted as a density heatmap without being normalized to DAPI. Whole slice normalizations across the mediolateral axis (all positive events for a gene in a given slice relative to DAPI) were plotted as bar graphs. DAPI and marker of interest counts are available in data S1 and S2.

### Neocortical areal proportions

In addition to the mediolateral subdivisions of marmoset neocortex, we further parcellated the neocortex into subregions referencing the Brain/MINDS 3D Digital Marmoset Atlas (table S7) (*76*). Slice selection was based upon visual recognition of prominent landmarks (white matter, striatal boundary, as well as DAPI nuclei staining) within our tissue and matched with the nearest atlas slice. ROIs for each parcellated region were created using the StrataQuest software in the anterior-posterior and dorsal-ventral axes (Fig. 6F). The corresponding feature selection that was previously set within the full neocortical sections was carried across gene and slice. The parameters used were mean intensity, maximum intensity, SD of intensity, range of intensity, and equivalent diameter. These filters were then applied to the unlabeled data to identify positive cells within each individual ROI. The parcellated neocortical areas were then analyzed using in-house code (https://doi.org/10.5281/zenodo.8364941; table S7). As above, positive events for a gene in a given ROI were normalized to DAPI and plotted in a (stacked) bar graph. Relative percentages between genes were calculated and plotted stacked.

### Morphology and smFISH experiments

AAV9-hDlx5/6-GFP-fGFP virus was generated as in (*29*). Virus was systemically intravenously injected (400 to 700 μl at 1.7 × 10^10^ to 2.4 × 10^10^ titers) in five marmosets. The virus was allowed to incubate for approximately 2 months. After systemic intravenous injection with AAV9-hDlx5/6-GFP-fGFP, marmosets were euthanized and perfused with saline followed by 4% PFA. Brains were removed, and 120-μm sections were cut on a vibratome into PBS-azide and stored at 4°C or moved into 70% ethanol for storage at −20°C. The 70% ethanol storage prevents RNA degradation at this temperature without considerable tissue shrinkage for short storage times. Because of laboratory shutdowns during the pandemic, sections from two marmosets were stored in 70% ethanol for approximately 4 months. These samples exhibited considerable shrinkage, measured by DAPI-stained nuclei diameters (tables S5 and S6); therefore, we only compared morphology parameters within-donor. Sections were taken as needed, and in situ hybridization was performed with HCR antisense probes, following the generic sample in solution HCR protocol with a twofold increase in concentration of probe to hybridization buffer (Molecular Instruments, https://files.molecularinstruments.com/MI-Protocol-RNAFISH-GenericSolution-Rev8.pdf; table S7), for markers of interest that corresponded with RNA-seq defined clusters. The sections were then stained with anti-GFP antibody and a secondary antibody (table S4) to amplify the endogenous GFP signal (https://www.protocols.io/view/marmoset-nhp-free-floating-anti-gfp-antibody-stain-3byl47nb2lo5/v1; table S7). Sections were incubated in TrueBlack (Biotium, 23007) for 3 to 5 min to mask confounding lipofuscin autofluorescence throughout the section. Sections were then mounted onto a slide and coverslipped with ProLong Diamond Antifade Mountant (Invitrogen, P36970) for imaging.

#### Imaging for morphology

Sections prepared for morphology were imaged on a Nikon Ti Eclipse inverted microscope with an Andor CSU-W1 confocal spinning disc unit and Andor DU-888 EMCCD using a 40×/1.15 NA water-immersion objective, and later on a TissueGnostic TissueFAXS SL slide-scanning, spinning disk confocal microscope (with Hamamatsu Orca Flash 4.0 v3) using a 40×/1.2 NA water-immersion objective. With the TissueFAXS, overview images were taken to select GFP^+^ cells for imaging at 40× and to highlight the exact location of the cell. GFP^+^ cells were imaged for stained markers of interest. Selected sections were imaged on an upright confocal laser scanning microscope (Olympus Fluoview FV3000) using a 40×/0.95 NA air objective or a 60×/1.50 NA oil-immersion objective and cooled Gallium Arsenide Phosphide Photomultiplier Tubes (GaAsP PMTs).

#### Morphological reconstruction and feature quantification - Imaris

Without preprocessing the confocal images, three-dimensional (3D) reconstruction and surface rendering of striatal and neocortical interneurons were performed using Imarisx64 9.9 software (Oxford Instruments) based on GFP^+^ signal. Surface-rendered images were used to determine the soma diameter, total volume, and total surface area for each z-stack image. 3D-skeleton diagrams (Figs. 8, A to C, and 9, H and J), corresponding to each surface-rendered image, were generated using the Filament Tracing wizard in Imaris and then pseudo-colored in Adobe Illustrator. The total number of primary dendritic branches, dendritic branch points, area, volume, and length of the 3D-skeleton diagrams was calculated using the AutoPath (no loops) algorithm in the filament tracing wizard in Imaris. The total number of primary dendritic branches for each cell is defined by the number of dendrite branches in the filament trace one distance value away from the soma. The distance value is calculated automatically by the AutoPath (no loops) algorithm based on the diameter of the soma and the diameter of the thinnest cellular process. All data were exported to CSV, and data collected from exemplar cells (Figs. 8, A and B, and 9G) were reported in table S6.

A separate dataset containing GFP^+^ interneurons from one animal (Cj 17-154; table S1) was first blinded using a custom Python script and then reconstructed with Imaris (Fig. 8, C and D). All results are presented as mean ± SEM. Comparisons between soma diameter, surface area, surface volume, and the number of primary branches were carried out using unpaired *t* tests. Unpaired Mann-Whitney *U* tests were used to statistically compare the total length, area, volume, and total number of dendritic branch point measurements. Statistically significant analyses were denoted as follows: *P < 0.05; **P < 0.01.

#### Morphological reconstruction and feature quantification - NeuTube

Automatic or semi-automatic tracing algorithms are challenged by some data, perhaps due to the low signal-to-noise ratio (SNR) of a given image. To overcome this, we manually reconstructed GFP+ neurons via Neutube (*114*) tracing software (table S5). With the software, we (i) create 3D volume rendering of the GFP-AAV marmoset neuron, (ii) use the signal transfer function (e.g., histogram equalization) for overall intensity and opacity values to optimize the SNR by manually examining the clearest visualization of the dendrites, (iii) build the neuron skeleton over the 3D volume by tracing the processes, (iv) scan through the 3D volume to make sure no parts of the neuron are missed, (v) check the raw 2D images to see if any of the branches were not presented well in 3D due to volume rendering artifacts, and (vi) label axon, dendrites, and soma parts of the skeleton model. Reconstructed neurons were saved as SWC format.

### snATAC-seq, library preparation, sequencing

snATAC-seq (10x Genomics Single Cell ATAC v1) was conducted on fresh marmoset tissue (1 female, 2 years old) dissected from anterior striatum. Nuclei suspensions were generated following the 10x Genomics recommended protocol (CG000212 Rev. B). Library preparation followed 10x Genomics Single Cell ATAC v1 Guide (CG000168 Rev. D). Library was sequenced on an Illumina NovaSeq (RRID:SCR_016387) using 100-bp paired-end reads to a median per-cell fragment depth of 25,472. Alignment and fragment counting was conducted using Cell Ranger (RRID:SCR_01734), aligned to marmoset genome cj1700 (https://www.ncbi.nlm.nih.gov/assembly/GCF_009663435.1/; GCA_009663445.2). snATAC-seq data were integrated with marmoset striatal snRNA-seq data from two independent animals (bi005, bi006) using Signac (RRID:SCR_021158) with the following parameters: integration method: cca; weight.reduction = lsi. Differentially accessible peaks for each cluster were calculated with a threefold change cutoff and the minimum fraction of expressed cells (in target cluster) = 0.2. Enhancer locations were as follows (CJ1700 coordinates): Eh14 = chr1-122696345-122696560 (215 bp); Eh15 = chr17-32919427-32919564 (137 bp); Eh16 = chr20-38770509-38770619 (110 bp); Eh17 = chr9-60258694-60258788 (94 bp).

### TAC3-AAV viral design and production

AAVs were produced in accordance with the protocol (*115*). Construct DNA (5.7 μg/150-mm dish) was transfected with pAAV9 capsid plasmid (22.8 μg/150-mm dish) and pAdDeltaF6 helper plasmid (11.45 μg/150-mm dish) using polyethylenimine 25K MW (Polysciences, 23966-1). Collection of cells and media for AAV harvesting began 72 hours following transfection before iodixanol gradient ultracentrifugation purification using a Type 70 Ti Fixed-Angle Titanium Rotor (Beckman-Coulter, 337922). Titer was calculated using digital droplet polymerase chain reaction (ddPCR) according to the protocol “ddPCR Titration of AAV Vectors” (https://www.addgene.org/protocols/aav-ddpcr-titration/) using the QX200 AutoDG Droplet Digital PCR System (Bio-Rad, 1864100). gBlock fragments containing the tandem *TAC3* enhancers (E14E15E16E17) were synthesized by Integrated DNA Technologies (IDT). The enhancers were cloned into AAV plasmid vector backbones containing a minimal cytomegalovirus (CMV) promoter, the reporter, and a barcode unique to each enhancer sequence using the NEBuilder HiFi DNA Assembly Cloning Kit (NEB-E5520S), following the standard protocol. Two microliters of Gibson assembly product was used to transform 50 μl of homemade Stbl3 cells following a standard transformation protocol. Mini-preparation of plasmids was carried out using the NucleoSpin mini kit (Macherey-Nagel). Positive clones were identified by restriction enzyme digestion and sequencing. The positive clones were grown in 300 ml of LB cultures, and the plasmids were extracted using the NucleoBond Xtra Midi EF kit (Macherey-Nagel). The plasmids were sent out for sequencing again before being packaged into AAVs.

### TAC3-AAV local injection procedure

#### Structural MRI scanning

In preparation for MR imaging under anesthesia, an animal (two marmosets, Cj 19-207 and 17-B111; table S1) checked for robust health was fasted overnight with ad libitum access to water. Before scanning, the animal was sedated with alfaxalone (5 to 10 mg/kg) or a combination of alfaxalone (4 to 8 mg/kg) and ketamine (5 to 10 mg/kg) given intramuscularly. It was then transferred to a custom-designed MRI cradle equipped with a 3D-printed nose cone for delivering anesthetic gases. The animal’s head was securely held to minimize motion artifacts and stereotaxically align the rostral-caudal axis with the MR scanner bore using contrast-filled MR-compatible ear bars, an adjustable palette bar, and eye bars. During scanning, anesthesia was maintained at 1 to 2% isoflurane in a mixture of oxygen. A temperature-controlled water circulating heat pad was used for thermal support. Heart rate and blood oxygen saturation levels were continuously monitored and recorded every 10 min with an MR-compatible monitoring system (Nonin, MN). MR scans consisted of high-resolution 3D T1 mapping using a magnetization-prepared rapid gradient-echo (MPRAGE) sequence (*116*) with repetition time (TR) = 6000 ms, TE (echo time) = 4.6 ms, flip angle (FA) = 12°, FOV = 80, matrix size = 256 × 256. A high-resolution T2-weighted anatomical scan was also obtained using a RARE pulse sequence with effective TE = 35.5 ms, TR = 2500 ms, RARE factor = 8, spatial resolution = 156 μm × 156 μm × 0.5 mm, matrix size = 256 × 256, RARE factor = 4, FOV = 80 mm.

#### Virus injection surgery

Following sedation with alfaxalone (5 to 10 mg/kg), the animal was intubated and maintained at 1.5 to 2% isoflurane in a mixture of oxygen throughout the rest of the procedure. An intravenous catheter was placed in the saphenous vein for infusion of fluids and drugs during the surgery and the recovery period. Heart rate, SpO_2_, electrocardiography, end-tidal CO_2_, and rectal temperature were continuously monitored and logged every 5 min throughout. Once the animal acquired a stable plane of anesthesia, it was placed in a stereotaxic apparatus (Narishige, Japan). A thin layer of sterile eye lubricant was applied to protect against corneal drying. Animal was also provided with a single bolus dose of intravenous dexamethasone (0.4 mg/kg) to guard against brain swelling. The scalp and fascia were removed in layers via blunt dissection to expose the injection sites. Using MRI atlas guidance, bilateral craniotomies were performed to expose the medial anterior caudate and ventral posterior putamen region. The tandem enhancer virus AAV9-tandemE-TAC3-EGFP was injected in left caudate (high titer, 10^13^; low volume, 0.5 μl) and left putamen (low titer, 10^11^; high volume, 1.5 μl). Each injection was delivered with a controlled syringe pump (100 nl/min). After each injection, the needle was kept in place for 10 min and then slowly retracted over 1 to 2 min to minimize injection backflow. Following withdrawal, the cranial openings were covered with bonewax, and fascia and scalp were sutured in layer-wise manner with 3-0 vicryl sutures. The suture site was cleaned with warm sterile saline and covered with hypafix. For recovery, the animal was transferred in an incubator and monitored closely until it was able to move effortlessly and accepted treats before returning to its home cage. Post-op medications were provided under veterinary advice for pain and inflammation control until full recovery. The animals were euthanized 6 to 10 weeks after viral injection and were perfused transcardially with 4% PFA. The brain was extracted for histology, smFISH, and confocal microscopy.

### TAC3-AAV-BI103 systemic injection procedure

One 2.5-year-old female marmoset (table S1) was sedated with alfaxalone (5 to 10 mg/kg) given intramuscularly. A catheter was placed into the tail vein, and 200 μl of purified viral particles was injected at a dose of 7.85 × 10^13^ viral genomes (vg)/kg, followed by injection of 1.5 ml of saline to flush the line. The animal was euthanized 4 weeks after viral injection and was perfused transcardially with 4% PFA. The brain was extracted for histology, smFISH, and confocal microscopy.

### TAC3-AAV histology

For AAV9-tandemE-TAC3-EGFP experiments, coronal sections around the injection sites were cut at 100 μm on a vibratome (Leica, VT1000S) and then stored in PBS-NaN_3_ at 4°C until use. For AAV-BI103-tandemE-TAC3-EGFP experiments, coronal and sagittal sections were processed. Sections were stained for *TAC3* (Molecular Instruments, PRC843; table S4) using the HCR v.3.0 protocol (Molecular Instruments) described above and a rabbit ​​anti-GFP antibody (Invitrogen, A11122; table S4) with subsequent secondary goat anti-rabbit conjugated with AF488 (Thermo Fisher Scientific, A-11008). Stained sections were then incubated for 1 to 3 min in TrueBlack to eliminate confounding lipofuscin autofluorescence. The striatum was imaged for each section using the TissueFAXS SL with a 40×/1.2 NA water-immersion lens through the whole thickness of the tissue.
