# Supplementary material for: A marmoset brain cell census reveals regional specialization of cellular identities
Source: Sci Adv. 2023 Oct 12;9(41):eadk3986. doi: 10.1126/sciadv.adk3986 (PMC10569717; doi:10.1126/sciadv.adk3986)
Supplement: Supplementary file 1 — Figs. S1 to S9 Legends for tables S1 to S7 Legends for data S1 and S2 [file sciadv.adk3986_sm.pdf]

Science Advances
AAAS

Supplementary Materials for

A marmoset brain cell census reveals regional specialization of cellular identities

Fenna M. Krienen *et al.*

Corresponding author: Fenna M. Krienen, fkrienen@princeton.edu; Steven A. McCarroll, mccarroll@genetics.med.harvard.edu; Guoping Feng, fengg@mit.edu

Sci. Adv. **9**, eadk3986 (2023)
DOI: 10.1126/sciadv.adk3986

The PDF file includes:

Figs. S1 to S9
Legends for tables S1 to S7
Legends for data S1 and S2

Other Supplementary Material for this manuscript includes the following:

Tables S1 to S7
Data S1 and S2

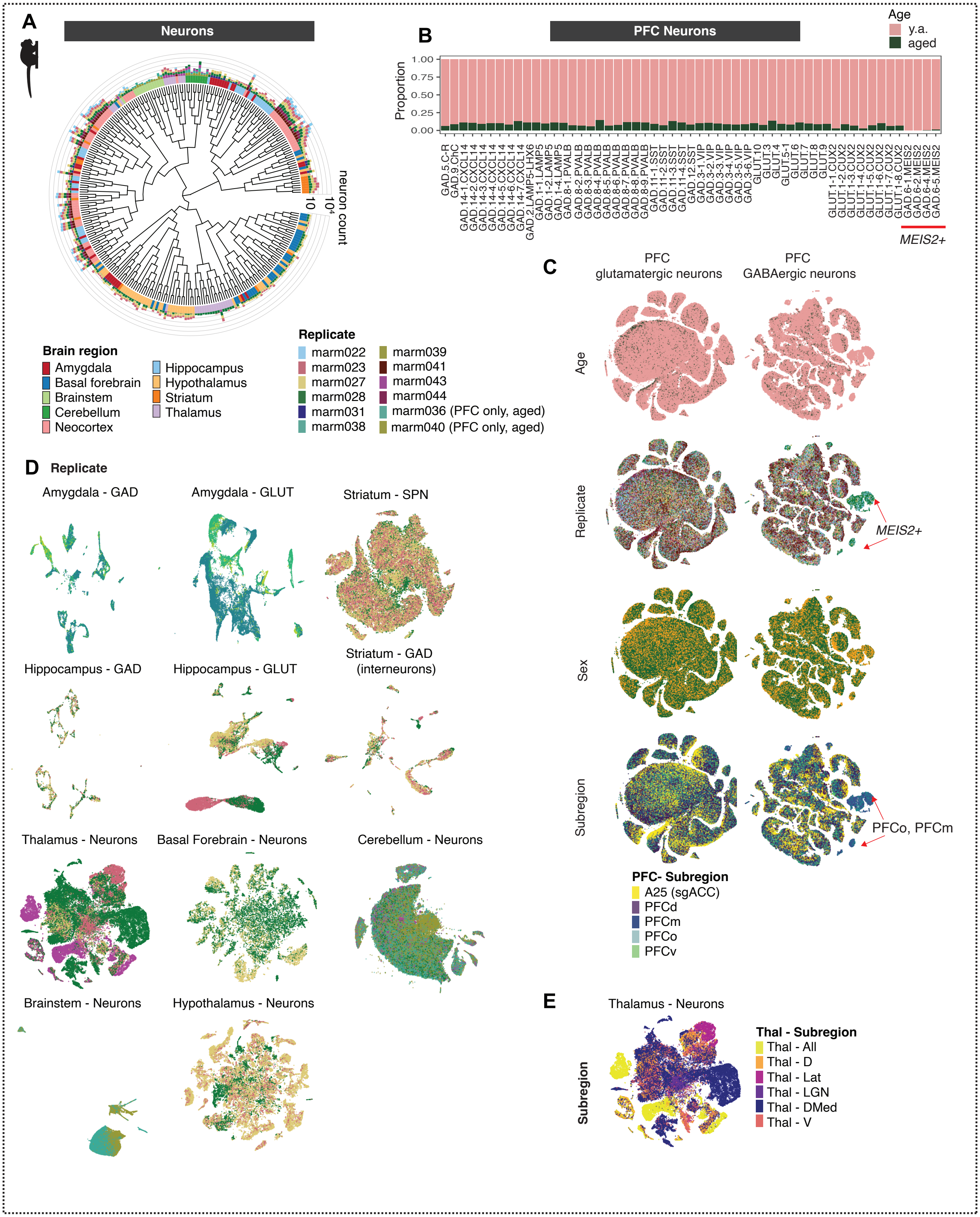
A
Neurons
neuron count
10
10^4
Brain region
Amygdala
Basal forebrain
Brainstem
Cerebellum
Neocortex
Hippocampus
Hypothalamus
Striatum
Thalamus
Replicate
marm022
marm023
marm027
marm028
marm031
marm038
marm039
marm041
marm043
marm044
marm036 (PFC only, aged)
marm040 (PFC only, aged)
B
PFC Neurons
Age
y.a.
aged
Proportion
1.00
0.75
0.50
0.25
0.00
GAD.5.C-R
GAD.9.ChC
GAD.14-1.CXCL14
GAD.14-2.CXCL14
GAD.14-3.CXCL14
GAD.14-4.CXCL14
GAD.14-5.CXCL14
GAD.14-6.CXCL14
GAD.14-7.CXCL14
GAD.2.LAMP5-LHX6
GAD.1-1.LAMP5
GAD.1-2.LAMP5
GAD.1-4.LAMP5
GAD.8-1.PVALB
GAD.8-2.PVALB
GAD.8-3.PVALB
GAD.8-4.PVALB
GAD.8-5.PVALB
GAD.8-6.PVALB
GAD.8-7.PVALB
GAD.8-8.PVALB
GAD.8-9.PVALB
GAD.11-1.SST
GAD.11-2.SST
GAD.11-3.SST
GAD.11-4.SST
GAD.12.SST
GAD.3-1.VIP
GAD.3-2.VIP
GAD.3-3.VIP
GAD.3-4.VIP
GAD.3-5.VIP
GAD.3-6.VIP
GLUT.10
GLUT.3
GLUT.4
GLUT.5-1
GLUT.6
GLUT.7
GLUT.8
GLUT.9
GLUT.1-1.CUX2
GLUT.1-2.CUX2
GLUT.1-3.CUX2
GLUT.1-4.CUX2
GLUT.1-5.CUX2
GLUT.1-6.CUX2
GLUT.1-7.CUX2
GLUT.1-8.CUX2
GAD.6-1.MEIS2
GAD.6-2.MEIS2
GAD.6-4.MEIS2
GAD.6-5.MEIS2
MEIS2+
C
PFC glutamatergic neurons
PFC GABAergic neurons
Age
Replicate
MEIS2+
Sex
Subregion
PFCo, PFCm
PFC- Subregion
A25 (sgACC)
PFCd
PFCm
PFCo
PFCv
D
Replicate
Amygdala - GAD
Amygdala - GLUT
Striatum - SPN
Hippocampus - GAD
Hippocampus - GLUT
Striatum - GAD (interneurons)
Thalamus - Neurons
Basal Forebrain - Neurons
Cerebellum - Neurons
Brainstem - Neurons
Hypothalamus - Neurons
E
Thalamus - Neurons
Subregion
Thal - Subregion
Thal - All
Thal - D
Thal - Lat
Thal - LGN
Thal - DMed
Thal - V

Fig. S1. Neuron counts by donor across brain regions. (A) Neuronal dendrogram as in **Fig. 1C**, with outer barplots depicting number of nuclei per cell type and replicate. Ring colors are brain regions, colors in barplots correspond to replicates. **(B)** Proportional per-cluster representation of PFC neurons between young adult donors and aged (n=2) donors. While our snRNA-seq collection focused on post-sexual maturity young adults, we acquired an additional dataset of PFC sampled from 2 aged animals (1 M, 11y5m; 1F, 14y5m, 37,260 cells total; **Table S1**). Individual replicates contributed similar proportions of neurons to each prefrontal neuron subtype, and clusters generally had proportional representation across young adults and aged animals, as well as across males and females, suggesting that these variables do not dramatically impact neuronal ensembles and identities in prefrontal cortex. **(C)** t-SNE embeddings of PFC neurons (*top row*, GABAergic; *bottom row*, glutamatergic) with colors representing different metadata: age (young vs aged), replicate, PFC subregion. There was notable enrichment of *MEIS2+* GABAergic neurons in medial prefrontal and orbital prefrontal dissections. Based on their gene expression profiles, these cells likely correspond to the recently described population of LGE-derived *MEIS2*+ neurons that populate the olfactory bulb in mice, and which are routed to medial prefrontal cortex in macaques and humans*(46)*. **(D)** t-SNEs of neurons in each brain structure, with cells colored by replicate (colors as in (A)). Telencephalic neurons are plotted separately by class: GABAergic and glutamatergic classes (neocortex, hippocampus, amygdala), or GABAergic interneurons and spiny projection neurons (striatum). Compared with neocortex, greater cross-donor variability was observed in some subcortical structures such as hypothalamus and thalamus, though this was likely driven more by dissection variability than by donor variability, as the donor-specific clusters tended to be from subregions that were only sampled in one individual (see E). **(E)** t-SNEs of thalamic neurons with cells colored by thalamic subdivision.

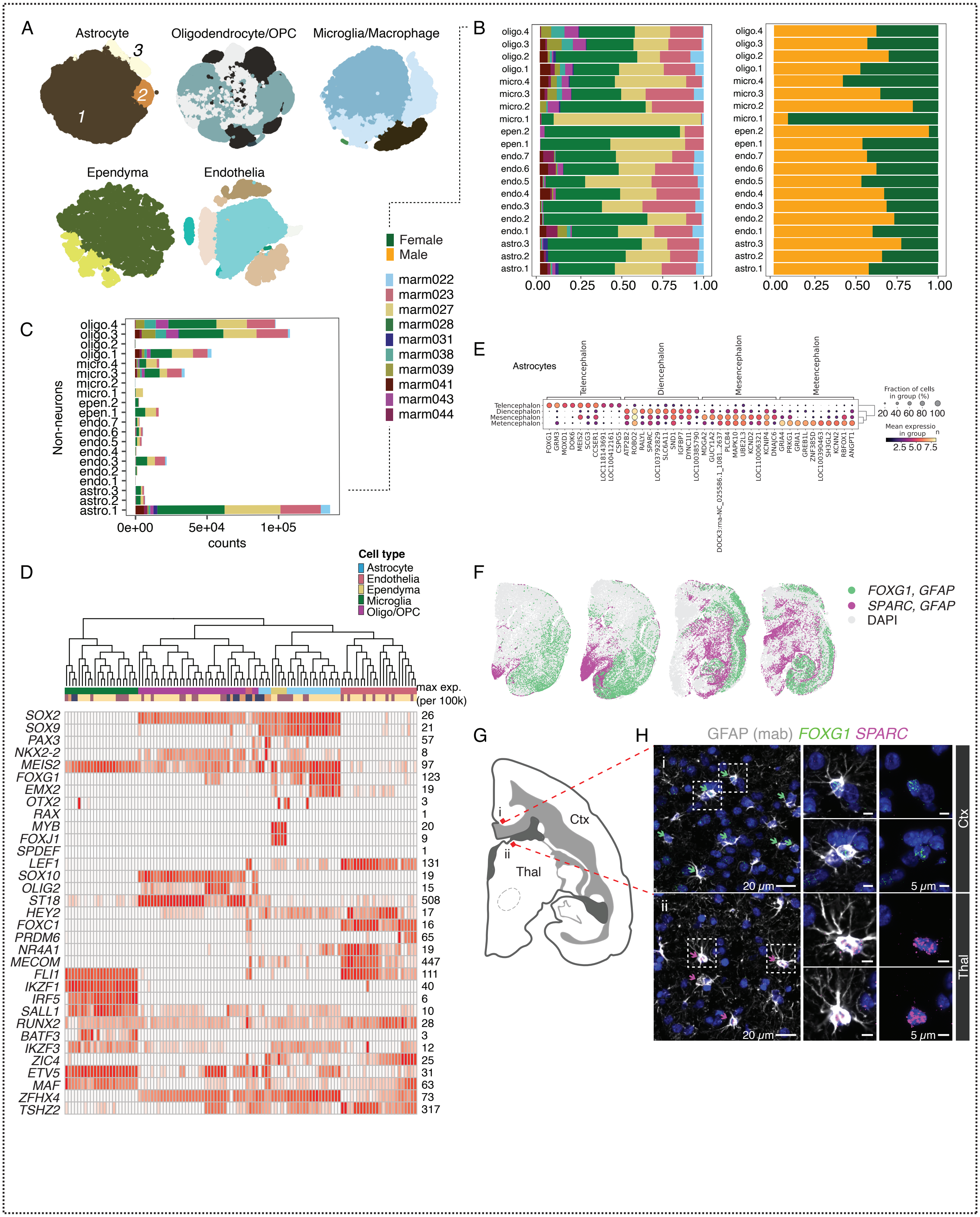
A
Astrocyte
Oligodendrocyte/OPC
Microglia/Macrophage
Ependyma
Endothelia
1
2
3
B
oligo.4
oligo.3
oligo.2
oligo.1
micro.4
micro.3
micro.2
micro.1
epen.2
epen.1
endo.7
endo.6
endo.5
endo.4
endo.3
endo.2
endo.1
astro.3
astro.2
astro.1
0.00 0.25 0.50 0.75 1.00
Female
Male
marm022
marm023
marm027
marm028
marm031
marm038
marm039
marm041
marm043
marm044
C
Non-neurons
0e+00 5e+04 1e+05
counts
D
Cell type
Astrocyte
Endothelia
Ependyma
Microglia
Oligo/OPC
max exp. (per 100k)
SOX2 26
SOX9 21
PAX3 57
NKX2-2 8
MEIS2 97
FOXG1 123
EMX2 19
OTX2 3
RAX 1
MYB 20
FOXJ1 9
SPDEF 1
LEF1 131
SOX10 19
OLIG2 15
ST18 508
HEY2 17
FOXC1 16
PRDM6 65
NR4A1 19
MECOM 447
FLI1 111
IKZF1 40
IRF5 6
SALL1 10
RUNX2 28
BATF3 3
IKZF3 12
ZIC4 25
ETV5 31
MAF 63
ZFHX4 73
TSHZ2 317
E
Astrocytes
Telencephalon
Diencephalon
Mesencephalon
Metencephalon
Fraction of cells in group (%)
20 40 60 80 100
Mean expression in group
2.5 5.0 7.5
F
FOXG1, GFAP
SPARC, GFAP
DAPI
G
Ctx
Thal
i
ii
H
GFAP (mab) FOXG1 SPARC
20 μm
5 μm
Ctx
Thal

Fig. S2. Glial diversity across brain structures. (A) t-SNE embeddings of major non-neuronal types colored by cluster. **(B)** Barplots of glial proportions colored by donor and by sex. **(C)** Non-neuronal nuclei counts. Colors indicate donor, same as (B). **(D)** Expression of marker genes in non-neurons. Genes as in*(1)*. Heatmap colors are scaled to max normalized expression for each row (gene). Dendrogram and metadata colors as in **Fig. 1D**. **(E)** Differentially expressed genes in astrocytes across cephalic compartments. **(F)** Tissue validation for astrocyte differentially expressed genes (*FOXG1, SPARC*) in coronal sections of marmoset brain. Green dots indicate locations of cells that stain positive for *GFAP* (IHC, mAb, **Table S4**) and *FOXG1* (smFISH). Magenta dots indicate cell positions for *GFAP* (IHC) and *SPARC* (smFISH). **(G)** Cartoon of coronal section imaged; Red boxes (*i-ii*) correspond to tissue validation in (H). **(H)** (*Left)* Fields of view from neocortex and thalamus stained for *GFAP* antibody (gray), *FOXG1* (green), and *SPARC* (magenta). Green arrows highlight *GFAP* cells colocalized with *FOXG1*, magenta arrows highlight *GFAP* cells colocalized with *SPARC.* (*Right*) Magnified examples of double positive cells in neocortex and thalamus. Ctx = cortex, Thal = thalamus.

A

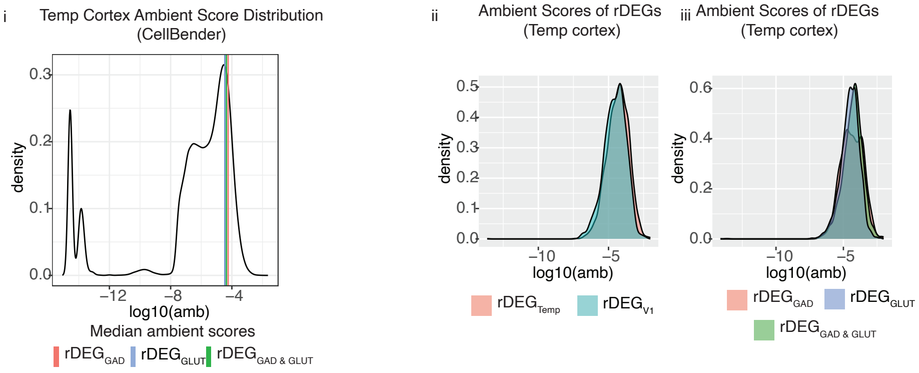

B

Neuron$_{GAD}$

	pfc	m1	temp	a1	s1	par	v2	v1
**pfc**	0	27	2	6	86	34	101	231
**m1**	5	0	5	1	1	2	2	24
**temp**	25	33	0	86	102	117	155	352
**a1**	16	22	13	0	77	70	97	190
**s1**	57	6	36	25	0	171	108	189
**par**	19	9	15	10	89	0	33	181
**v2**	27	1	36	10	18	5	0	54
**v1**	72	14	109	27	53	62	19	0

Neuron$_{GLUT}$

	pfc	m1	temp	a1	s1	par	v2	v1
**pfc**	0	118	65	136	202	292	401	413
**m1**	80	0	132	37	9	47	90	108
**temp**	96	242	0	28	96	173	321	364
**a1**	152	98	16	0	111	254	314	368
**s1**	154	35	55	44	0	272	308	358
**par**	131	36	87	53	105	0	74	280
**v2**	201	38	175	111	89	31	0	82
**v1**	211	57	189	120	150	141	58	0

Oligodendrocyte lineage

	pfc	m1	temp	a1	s1	par	v2	v1
**pfc**	0	39	13	3	65	17	81	90
**m1**	15	0	7	9	12	3	5	3
**temp**	37	57	0	41	103	47	25	41
**a1**	201	10	2	0	121	62	76	128
**s1**	186	5	3	14	0	79	88	99
**par**	153	2	1	16	121	0	10	99
**v2**	191	13	4	40	96	34	0	83
**v1**	141	6	9	37	84	45	33	0

Astrocytes

	pfc	m1	temp	a1	s1	par	v2	v1
**pfc**	0	71	23	29	149	42	72	151
**m1**	22	0	30	3	4	1	8	8
**temp**	44	38	0	12	32	24	51	50
**a1**	98	24	6	0	95	59	46	86
**s1**	104	3	12	21	0	86	55	83
**par**	221	19	11	57	204	0	95	248
**v2**	81	11	29	26	36	6	0	23
**v1**	134	14	43	44	68	53	14	0

Fig. S3. Cortical rDEGs do not reflect ambient RNA contamination and vary across region pairs. (A) *(i)* rDEG ambient score distributions (from CellBender *(111)*) in temporal cortex samples, which had amongst the highest numbers of rDEGs compared to other neocortical regions. Despite glutamatergic neurons being more numerous and having more expressed genes/ transcripts per cell, the median ambient contamination scores for glutamatergic rDEGs were not higher than median contamination scores for GABAergic rDEGs. rDEGs shared between glutamatergic and GABAergic neurons had indistinguishable scores compared with rDEGs private to one neuronal class. *(ii)* Ambient scores in temporal cortex of temporal cortex rDEGs are indistinguishable from V1 rDEGs. *(iii)* Distributions of temporal cortex rDEG ambient scores by neuron class, again showing no difference between rDEGs that are shared or private to a neuronal class. **(B)** Numbers of regionally differentially expressed genes (rDEGs) between pairs of cortical regions for neurons, astrocytes, and oligodendrocyte lineage types.

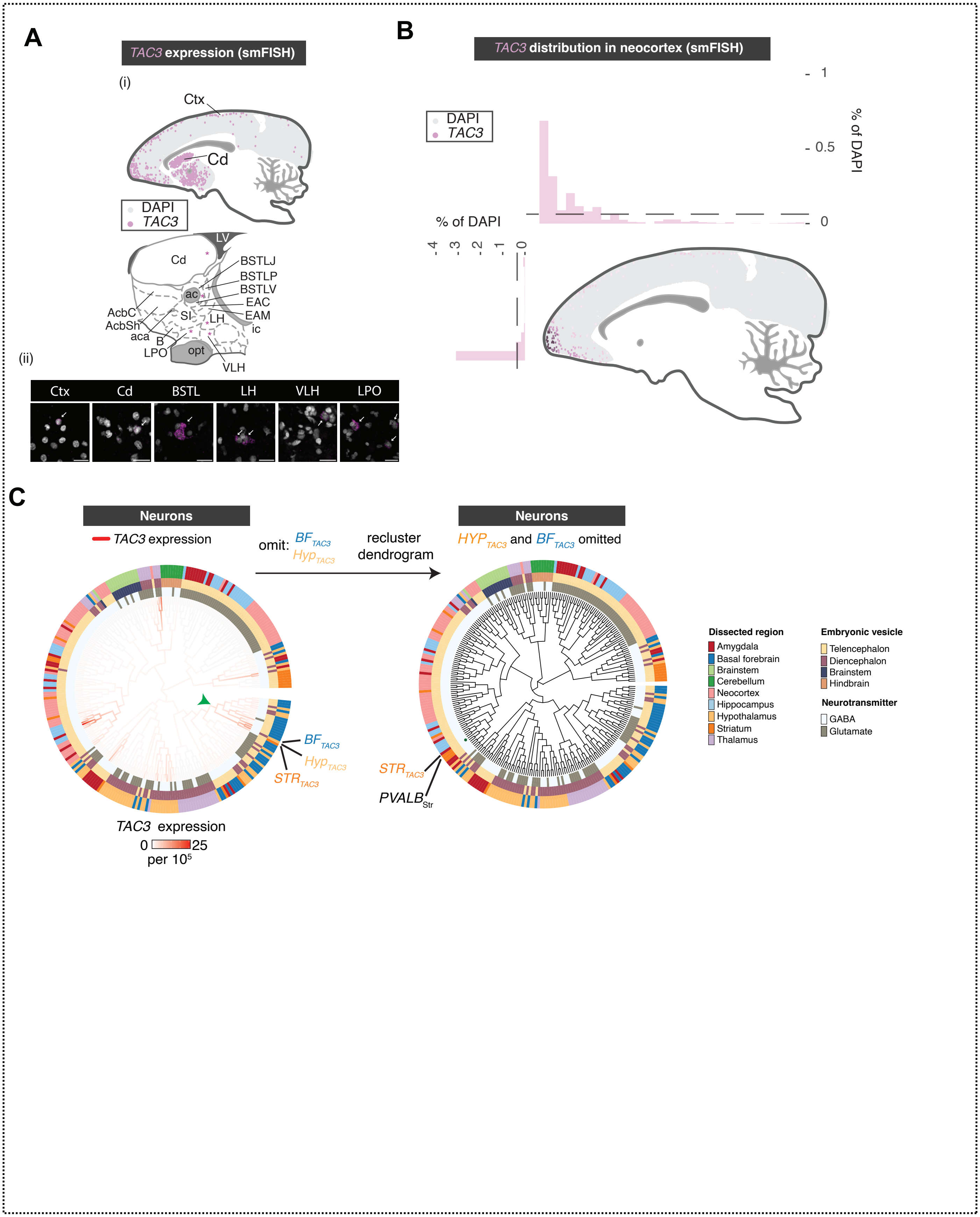
A
TAC3 expression (smFISH)
(i)
Ctx
Cd
DAPI
TAC3
LV
BSTLJ
BSTLP
BSTLV
ac
EAC
EAM
AcbC
AcbSh
SI
LH
ic
aca
B
LPO
opt
VLH
(ii)
Ctx
Cd
BSTL
LH
VLH
LPO
B
TAC3 distribution in neocortex (smFISH)
DAPI
TAC3
% of DAPI
1
0.5
0
-4
-3
-2
-1
0
C
Neurons
TAC3 expression
omit: BF TAC3 Hyp TAC3
recluster dendrogram
Neurons
HYP TAC3 and BF TAC3 omitted
BF TAC3
Hyp TAC3
STR TAC3
STR TAC3
PVALB Str
TAC3 expression
0
25
per 10⁵
Dissected region
Amygdala
Basal forebrain
Brainstem
Cerebellum
Neocortex
Hippocampus
Hypothalamus
Striatum
Thalamus
Embryonic vesicle
Telencephalon
Diencephalon
Brainstem
Hindbrain
Neurotransmitter
GABA
Glutamate

Fig. S4. Locations of *TAC3*+ cells in marmoset forebrain. (A) smFISH reveals anatomical locations and expression levels *TAC3+* types in different brain regions. *(i)* Schematic of *TAC3*+ cells imaged across cortex, dorsal striatum. Ctx = neocortex, Cd = Caudate. *(ii)* Cartoon close-up of nuclei in striatum, basal forebrain and hypothalamus. Magenta stars = locations of *TAC3*+ cells in lower image panel. Cd = Caudate, AcbC = nucleus accumbens core, AcbSh = nucleus accumbens shell, SI = Substantia innominata, B = basal nucleus of Meynert, EAM = extended amygdala, medial, EAC = extended amygdala, central, ac = anterior commissure, BSTLP = bed nuc st, lateral posterior, BSTLJ = bed nuc st, juxtacap, BSTLV = bed nuc st, lateral ventral, LH = lateral hypothalamus, VLH = ventrolateral hypothalamus, LPO = lateral preoptic area. **(B)** Density and location of *TAC3*+ cells as proportion of all DAPI+ cells. Barplots show percentages in bins (approximately 1,290 µm per bin) taken across the anterior-posterior (top) and dorsal-ventral (left side) axes. **(C)** Effect on placement of the *TAC3+* striatal type on the neuronal dendrogram when omitting the two *TAC3*+ types in hypothalamus and basal forebrain. When these types are omitted and hierarchical clustering is repeated (using HCA_{PCA}), the *TAC3*+ striatal type is most similar to *PVALB*+ striatal interneurons, consistent with previous reports that only compared telencephalic interneurons(*10*).

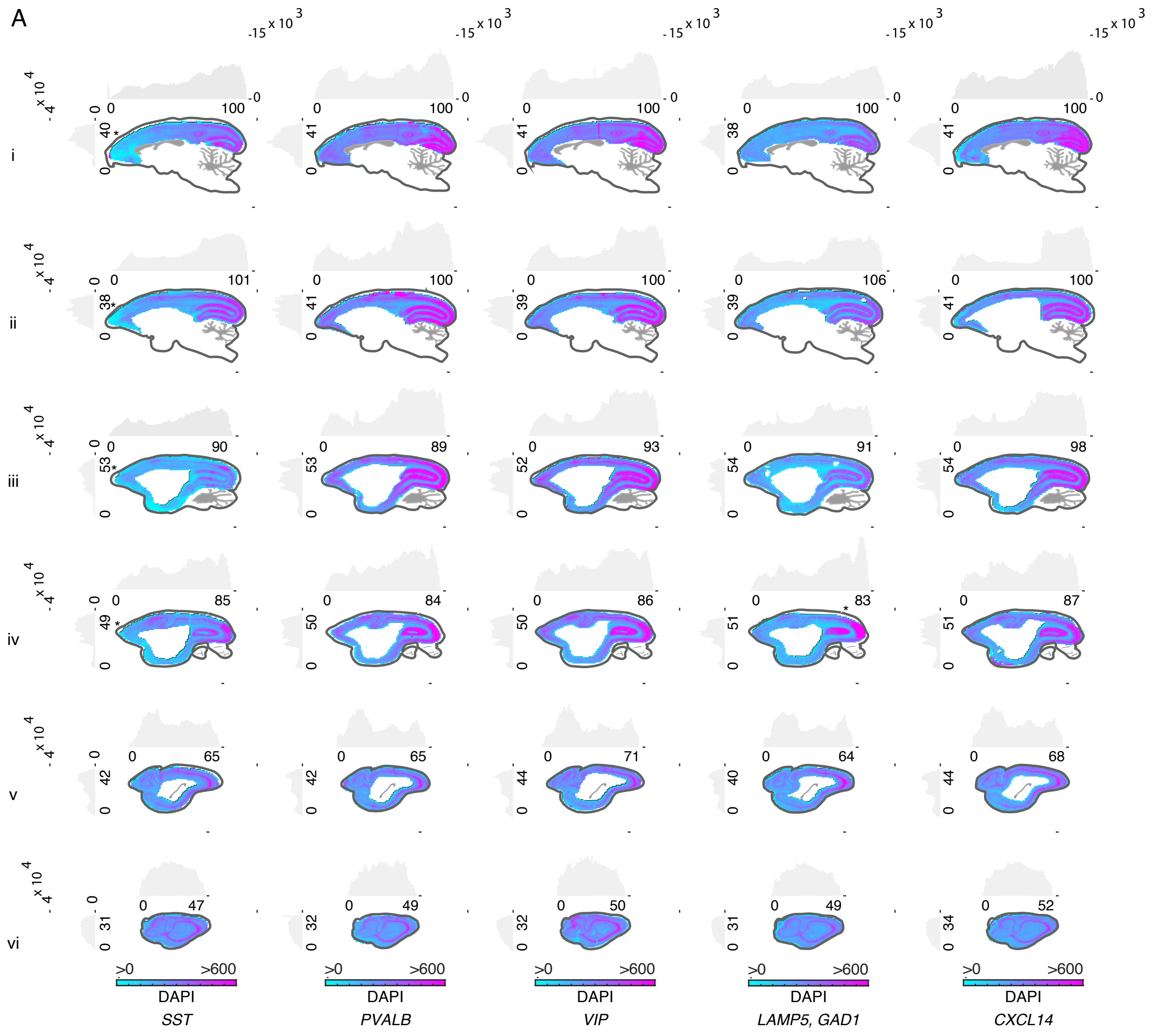

A
i
ii
iii
iv
v
vi
>0
>600
DAPI
SST
PVALB
VIP
LAMP5, GAD1
CXCL14

Fig. S5. Estimated cell numbers across marmoset neocortex. (A) Total numbers of DAPI+ cells per unit area (approximately 387 µm per bin) for each of the sections shown in **Fig. 6C.**

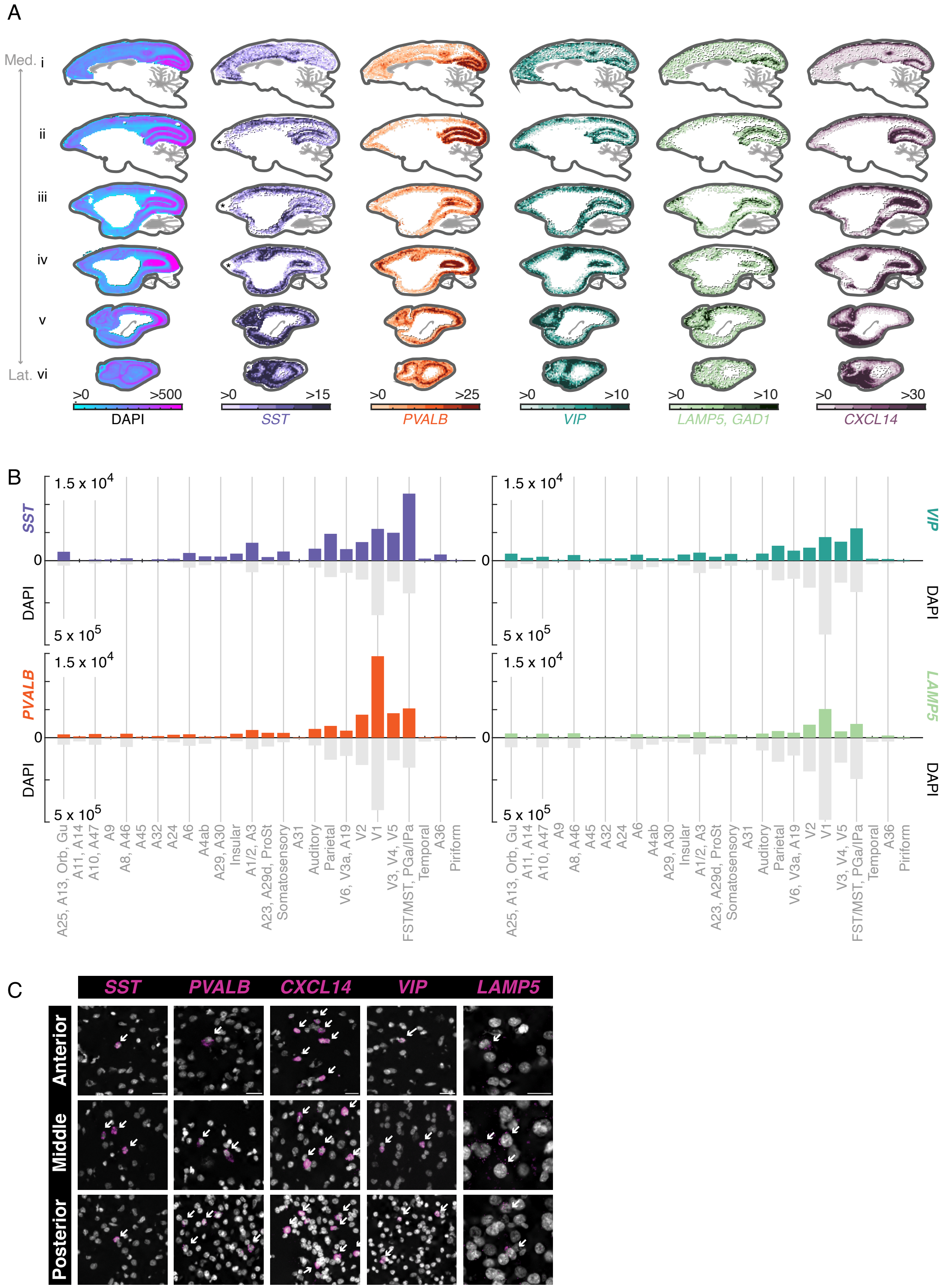

A
Med.
Lat.
i
ii
iii
iv
v
vi
>0 >500 DAPI
>0 >15 SST
>0 >25 PVALB
>0 >10 VIP
>0 >10 LAMP5, GAD1
>0 >30 CXCL14
B
SST
VIP
PVALB
LAMP5
DAPI
1.5 x 10^4
5 x 10^5
0
A25, A13, Orb, Gu
A11, A14
A10, A47
A9
A8, A46
A45
A32
A24
A6
A4ab
A29, A30
Insular
A1/2, A3
A23, A29d, ProSt
Somatosensory
A31
Auditory
Parietal
V6, V3a, A19
V2
V1
V3, V4, V5
FST/MST, PGa/IPa
Temporal
A36
Piriform
C
SST
PVALB
CXCL14
VIP
LAMP5
Anterior
Middle
Posterior

Fig. S6. Estimated interneuron numbers across marmoset neocortex. (A) smFISH for neocortical interneuron subclass markers showing locations of cells positive for each marker across 6 sagittal sections of the marmoset neocortex. Heatmap scale shows absolute density per unit area (approximately 387 µm per bin). First column shows DAPI and area profiled. **(B)** Quantification of interneurons (*SST*, *PVALB, VIP*, and *LAMP5* (*GAD1+*)) and DAPI cells by cortical area in marmoset parcellated according to **Fig. 6F** (anterior to posterior). DAPI counts are plotted on the inverse *y*-axis. **(C)** Examples of smFISH images quantitated in **Fig. 6B-E**. Panels for each marker show example positive cells in anterior, middle, and posterior locations across neocortex. White arrows indicate positive cells. Scale bar = 20 µm.

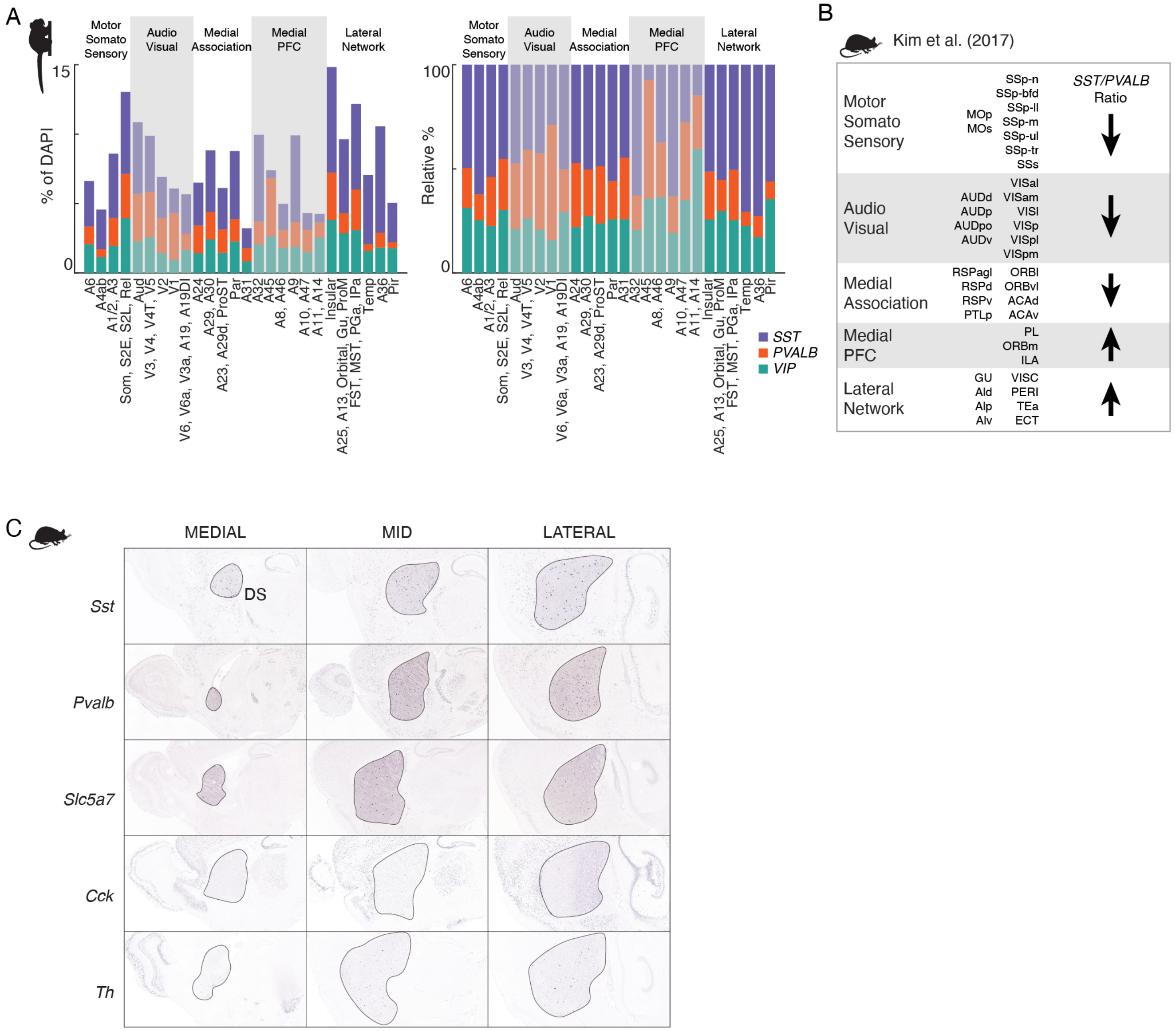
A
Motor Somato Sensory
Audio Visual
Medial Association
Medial PFC
Lateral Network
15
0
% of DAPI
100
0
Relative %
A6
A4ab
A1/2, A3
Som, S2E, S2L, Rel
Aud
V3, V4, V4T, V5
V2
V1
V6, V6a, V3a, A19, A19DI
A24
A29, A30
A23, A29d, ProST
Par
A31
A32
A45
A8, A46
A9
A10, A47
A11, A14
Insular
A25, A13, Orbital, Gu, ProM
FST, MST, PGa, IPa
Temp
A36
Pir
SST
PVALB
VIP
B
Kim et al. (2017)
SST/PVALB Ratio
Motor Somato Sensory
MOp
MOs
SSp-n
SSp-bfd
SSp-ll
SSp-m
SSp-ul
SSp-tr
SSs
Audio Visual
AUDd
AUDp
AUDpo
AUDv
VISal
VISam
VISl
VISp
VISpl
VISpm
Medial Association
RSPagl
RSPd
RSPv
PTLp
ORBl
ORBvl
ACAd
ACAv
Medial PFC
PL
ORBm
ILA
Lateral Network
GU
AId
AIp
AIv
VISC
PERI
TEa
ECT
C
MEDIAL
MID
LATERAL
DS
Sst
Pvalb
Slc5a7
Cck
Th

Fig. S7. Interneuron proportions in marmoset and mouse. (A) Quantitation of interneuron proportions by cortical area in marmoset parcellated according to **Fig. 6F**. **(B)** Schematization of interneuron proportions by cortical area in mouse reproduced from (*22*). **(C)** Mouse striatal *in situ* from Allen Brain Atlas for *Sst, Pvalb, Slc5a7, Cck, Th*; medial to lateral. DS = dorsal striatum.

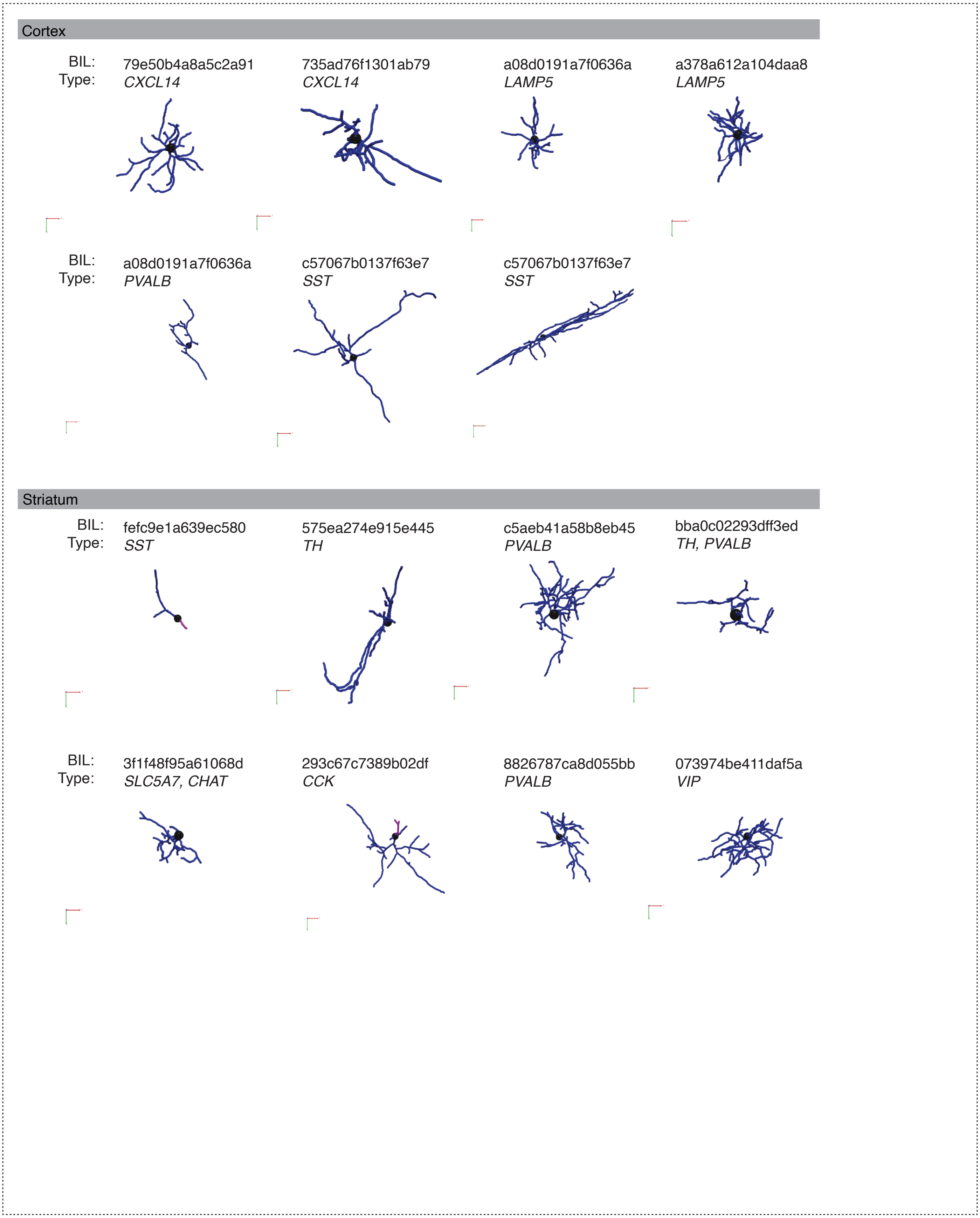
Cortex
BIL:
Type:
79e50b4a8a5c2a91
CXCL14
735ad76f1301ab79
CXCL14
a08d0191a7f0636a
LAMP5
a378a612a104daa8
LAMP5
BIL:
Type:
a08d0191a7f0636a
PVALB
c57067b0137f63e7
SST
c57067b0137f63e7
SST
Striatum
BIL:
Type:
fefc9e1a639ec580
SST
575ea274e915e445
TH
c5aeb41a58b8eb45
PVALB
bba0c02293dff3ed
TH, PVALB
BIL:
Type:
3f1f48f95a61068d
SLC5A7, CHAT
293c67c7389b02df
CCK
8826787ca8d055bb
PVALB
073974be411daf5a
VIP

Fig. S8. Morphology examples using NeuTube reconstructions. Example morphological reconstructions of striatal and neocortical interneurons using the NeuTube pipeline. Each cell, along with associated smFISH staining, is available for download at https://doi.org/10.35077/g.609.

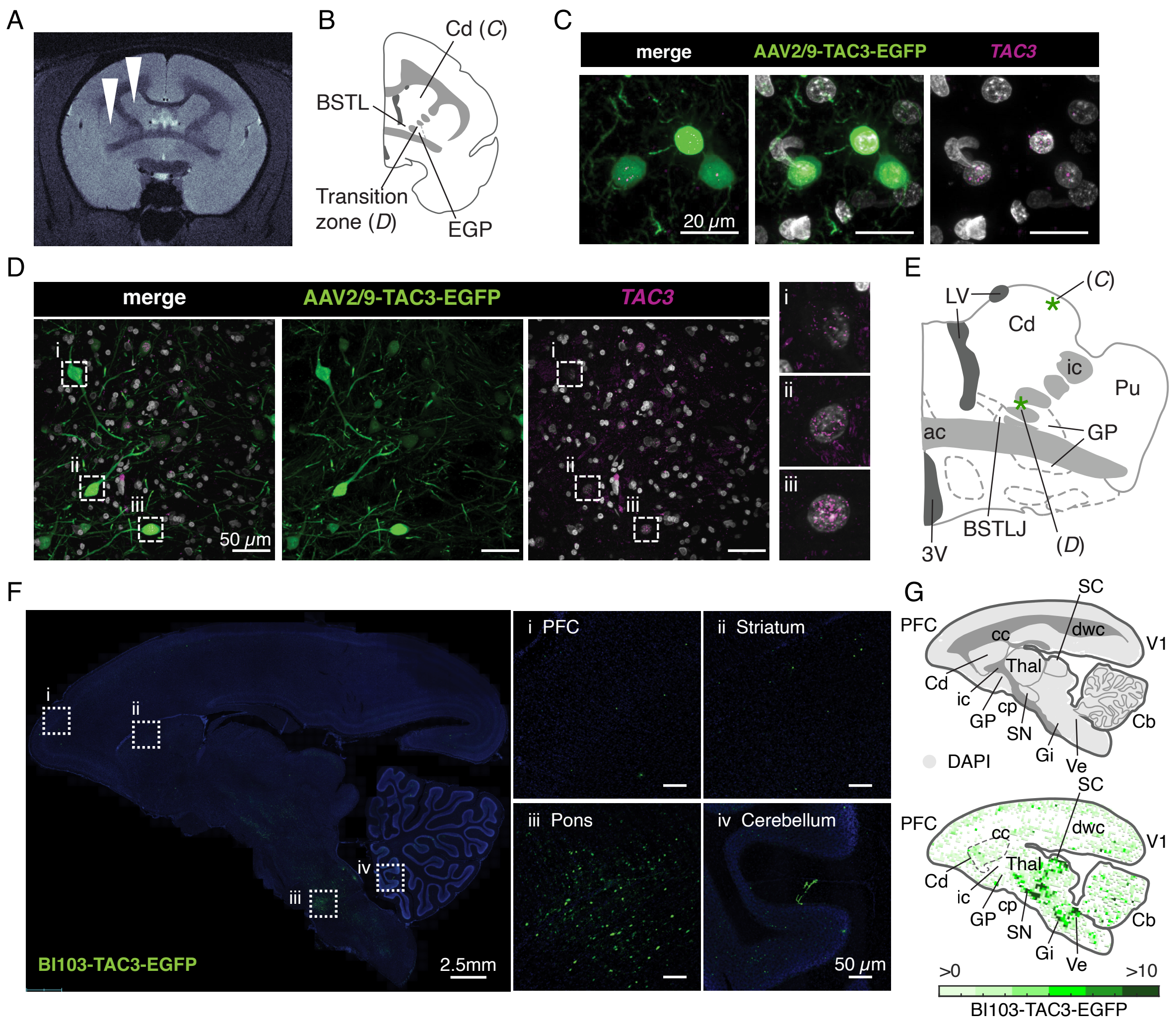

A
B
Cd (C)
BSTL
Transition zone (D)
EGP
C
merge
AAV2/9-TAC3-EGFP
TAC3
20 μm
D
merge
AAV2/9-TAC3-EGFP
TAC3
i
ii
iii
50 μm
E
LV
(C)
Cd
ic
Pu
ac
GP
3V
BSTLJ
(D)
F
i PFC
ii Striatum
iii Pons
iv Cerebellum
BI103-TAC3-EGFP
2.5mm
50 μm
G
SC
PFC
cc
dwc
V1
Thal
Cd
ic
cp
GP
SN
Cb
Gi
Ve
DAPI
>0
>10
BI103-TAC3-EGFP

Fig. S9. Examples of striatal and peri-striatal *TAC3*+ neurons labeled by AAV-tandemE-TAC3-EGFP. (A) MRI showing injection location (white arrowheads) of virus into unilateral striatum (caudate and putamen) in one animal (Cj 17-B111, **Table S1**). **(B)** Cartoon showing location of positive cells in (C) as well as labeled cells in transition zone (D). **(C)** EGFP antibody-amplified confocal image of a labeled cell (position shown in (B)) with smFISH for *TAC3* showing colocalization. Scale bar = 20 µm. **(D)** Examples of extra-striatal labeled cells from injections in (A). Position shown in (B). Scale bar = 50 µm. **(E)** Close up of position of cells in (C) and (D). Cd = caudate, Pu = putamen, ic = internal capsule, ac = anterior commissure, BSTLJ = bed nuc st, juxtacap, EGP = external globus pallidus, 3V = third ventricle, LV = lateral ventricle. **(F)** Whole sagittal section (20x image) of adult marmoset showing cells transduced by the AAV-BI103-tandemE-TAC3-EGFP virus in marmoset, Cj 20-214 (**Table S1**). EGFP+/*TAC3*+ cells were detected sparsely in neocortex as well as striatum, cerebellum, substantia nigra, superior colliculus, and brainstem. Scale bar = 2.5 mm. (*i*) Prefrontal cortex, (*ii*) Striatum, (*iii*) Pons, (*iv*) Cerebellum. Scale bar = 50 µm. **(G)** Heatmap showing the locational density of cells transduced by the AAV-BI103-tandemE-TAC3-EGFP virus across one sagittal section of the marmoset brain after systemic IV injection - (*top*) reference cartoon superimposed on a scatterplot of DAPI cells profiled (light gray) and (*bottom*) density plot of EGFP+ cells. Heatmap scale shows absolute density per unit area (approximately 344 µm per bin). PFC = prefrontal cortex, Cd = caudate, cc = corpus callosum, ic = internal capsule, GP = globus pallidus, Thal = thalamus, cp = cerebral peduncle, SN = substantia nigra, Gi = gigantocellular reticular nucleus, SC = superior colliculus, dwc = deep cortical white matter, Ve = vestibular nucleus, V1 = visual cortex V1, Cb = cerebellum.

Supplementary Table Legends (separate files)

Table S1. Marmoset sample information. Table of animals, metadata, and experimental information.

Table S2. snRNA-seq dataset by donor and brain area. Tables of the number of cells per brain structure and samples per donor, dendrogram of metacells, PC scores.

Table S3. Neocortical rDEGs across three donors. Neocortical rDEGs across three donors with pairwise comparisons between neocortical locations for major clusters of cortical excitatory neurons, inhibitory neurons, astrocytes and oligodendrocyte lineage types.

Table S4. Fluorescent *in situ* hybridization probes and antibodies used. A list of all FISH probes (RNA-Scope and Molecular Instruments) and antibodies used for validation of gene and protein expression *in situ*.

Table S5. Morphological reconstructions performed with Neutube. A list of all cells reconstructed with Neutube with their corresponding morphological measurements.

Table S6. Morphological reconstructions performed with Imaris. A list of all cells reconstructed with Imaris with their corresponding morphological measurements.

Table S7. Links and DOIs. A list of all links and DOIs referenced.

Supplementary Data Legends (separate files)

Data S1. *TAC3* medio-lateral quantification. Tabular data for *TAC3* medio-lateral histograms and barplots shown in **Fig. S4**.

Data S2. Striatal and cortical medio-lateral quantification. Tabular data for striatal and cortical medio-lateral histograms/bar plots shown in **Figs. 6-7**.
